# Functional identification of a feather-degrading thermitase-like S8 serine protease from *Fervidobacterium islandicum* AW-1

**DOI:** 10.3389/fmicb.2026.1886669

**Published:** 2026-08-11

**Authors:** Ji Seob Kim, Jae-Yoon Sung, Hyeon-Su Jin, Hakyoo Jeong, Gaewon Nam, Dong-Woo Lee

**Affiliations:** 1 Department of Biotechnology, Yonsei University, Seoul, Republic of Korea; 2 Graduate Program in Bioindustrial Engineering, Yonsei University, Seoul, Republic of Korea; 3 Bio-Living Engineering Major, Global Leaders College, Yonsei University, Seoul, Republic of Korea

**Keywords:** feather keratin, *Fervidobacterium islandicum* AW-1, keratin degradation, S8 serine protease, structure–function relationship, thermophile

## Abstract

Thermophilic species of *Fervidobacterium* are well known for their ability to degrade native chicken feathers, yet the specific proteases responsible for this activity at the single-enzyme level have remained unresolved because previously identified enzymes primarily acted on synthetic substrates or required synergistic activity in crude extracts. Here, we identified and characterized Fervitase, a feather-degrading thermitase-like S8 serine protease from *Fervidobacterium islandicum* AW-1. Recombinant Fervitase efficiently hydrolyzed native chicken feathers as a purified enzyme in the presence of dithiothreitol and exhibited maximal activity at 80 °C and pH 8.0. LC–MS/MS analysis demonstrated broad cleavage specificity with preferential recognition of threonine, leucine, and serine residues at the P1 position. Notably, Fervitase selectively degraded insoluble feather keratin while exhibiting minimal activity toward soluble protein substrates, suggesting evolutionary specialization for keratin degradation. Structural and mutational analyses further demonstrated that Fervitase-specific structural elements are essential for maturation, catalytic activity and thermostability. Together, this study resolves a long-standing ambiguity in *Fervidobacterium* keratinolysis and expands current understanding of functional adaptation in thermophilic S8 proteases.

## 1 Introduction

The global poultry industry generates enormous quantities of feather waste annually, with chicken feathers accounting for approximately 5–7% (w/w) of total body weight per bird (Manczinger et al., 2003). Worldwide feather production exceeds 13 million metric tons annually (Kumari and Kumar, 2020), and the accumulation of this waste contributes to soil and water pollution while increasing the risk of pathogen dissemination (Bhari et al., 2021). Despite these environmental concerns, feathers represent a highly concentrated and renewable protein resource composed of more than 90% keratin and enriched in sulfur-containing amino acids such as cystine, cysteine, and methionine (Sharma and Devi, 2017; Greenwold et al., 2014). Consequently, feather-derived hydrolysates have attracted considerable interest for applications in fertilizers (Możejko and Bohacz, 2023; Tamreihao et al., 2019), animal feed (Vidmar and Vodovnik, 2018), cosmetics (Jin et al., 2018; Yeo et al., 2018), and biodegradable biomaterials (Kim et al., 2026; Verma et al., 2016).

The remarkable recalcitrance of feather biomass primarily arises from *β*-keratin, a rigid fibrous biopolymer stabilized by extensive intra- and intermolecular disulfide and hydrogen bonding networks (Jin et al., 2018). These structural features confer exceptional mechanical resilience while simultaneously rendering feathers highly resistant to biodegradation, including hydrolysis by common proteases such as pepsin and trypsin (Ahn et al., 2011; Jin et al., 2017). Conventional feather-processing methods, including incineration, landfilling, and high-temperature chemical hydrolysis, are therefore energy-intensive and environmentally unsustainable, often causing destruction of essential amino acids, reduced digestibility, and generation of low-quality feather meal (Zhou et al., 2020; Cheong et al., 2018). Accordingly, there is increasing demand for efficient biocatalytic strategies capable of converting native keratin into soluble and functional biomaterials under environmentally compatible conditions (Kim et al., 2026).

Microbial keratinolysis has emerged as one of the most promising approaches for feather degradation (de Menezes et al., 2021; Qiu et al., 2022). Effective keratin decomposition generally requires initial reduction of disulfide bonds to destabilize the keratin scaffold, followed by proteolytic cleavage of the exposed peptide backbone by serine or metalloproteases (Kang et al., 2020). Numerous feather-degrading bacteria (FDB), including species of *Bacillus*, *Staphylococcus*, *Enterococcus*, *Streptomyces*, and *Pseudomonas*, have been identified (Li et al., 2020). However, most mesophilic FDB and their keratinases exhibit limited thermostability and require several days to efficiently degrade feather substrates, restricting their industrial applicability under elevated temperature and alkaline processing conditions (Zhang and Han, 2024; Peng et al., 2022).

In contrast, thermophilic *Fervidobacterium* species exhibit exceptional keratinolytic efficiency and can completely degrade native feathers within 48–72 h at temperatures above 60 °C (Nam et al., 2002; Ghasemi et al., 2011; Bhagya Jyothi and Dhanasingh, 2025). High-temperature processing enables direct utilization of untreated feathers while minimizing contamination by mesophilic microorganisms, making thermophilic keratinolysis particularly attractive for sustainable feather bioconversion. Consequently, thermostable keratinases from thermophiles have gained considerable attention as industrial biocatalysts because of their high catalytic efficiency and resistance to denaturing conditions such as detergents and organic solvents (Jin et al., 2017; Kang et al., 2020; Wang, 2019; Li, 2021; Wu et al., 2017). Nevertheless, many reported keratinases have primarily been evaluated using modified substrates such as keratin azure or azokeratin rather than intact native feathers, leaving their true keratinolytic capability unresolved (Riffel et al., 2003; Nnolim et al., 2020). Even representative thermostable keratinases such as MtaKer from *Meiothermus taiwanensis* WR-220 often fail to completely degrade feather rachis and barbs under practical processing conditions (Wu et al., 2017).

Previous studies from our group demonstrated that *Fervidobacterium islandicum* AW-1 encodes multiple proteases potentially involved in keratin degradation (Kang et al., 2020). However, the specific enzymes responsible for feather hydrolysis at the single-enzyme level have remained unresolved because multiple S8 serine proteases coexist within the genome and exhibit overlapping proteolytic features (Kang et al., 2020). Most candidate proteases displayed limited transcriptional induction during feather degradation (Sung et al., 2026), whereas the serine protease encoded by NA23_05775 exhibited approximately four-fold increased transcription during active keratinolysis (Kang et al., 2020), strongly implicating its involvement in feather degradation. Notably, several thermostable keratinases, including thermitase from *Thermoactinomyces vulgaris*, DgeKer from *Deinococcus geothermalis*, GacK from *Geoglobus acetivorans*, and islandisin from *F. islandicum*, belong to the S8 serine protease family and share conserved catalytic architectures despite substantial differences in substrate specificity and thermal adaptation (Tang et al., 2021; Bromme and Kleine, 1984; Sittipol et al., 2021; Godde et al., 2005). These observations suggest that subtle structural variations within the conserved S8 scaffold may critically influence keratin specificity and thermostability.

Although the crystal structure of a *Fervidobacterium* S8 peptidase FerB from *F. pennivorans* strain T has recently been solved at 1.5 Å resolution, in an auto-processed state, revealing a tyrosine-rich *β*-hairpin that contributes to structural integrity and keratinase activity (Krüger et al., 2026), the structural and functional determinants underlying efficient native-feather degradation, substrate specificity, and thermophilic keratinolysis remains largely unexplored in closely related feather-degrading *Fervidobacterium* species. Here, we functionally identified and characterized the NA23_05775-encoded enzyme, termed Fervitase, as a thermophilic feather-degrading S8 serine protease from *F. islandicum* AW-1. Through phylogenetic analysis, structural modeling, biochemical characterization, and mutational analyses, we investigated the structural basis underlying its keratinolytic activity and thermophilic adaptation. Our findings reveal that Fervitase possesses distinct structural elements essential for feather recognition and efficient keratin degradation, providing new insight into the functional specialization of thermophilic S8 proteases.

## 2 Materials and methods

### 2.1 Sequence and phylogenetic analyses

Phylogenetic and sequence analyses were performed to evaluate the evolutionary relationship of Fervitase (NA23_05775) from *F. islandicum* AW-1 with homologous S8 proteases. Full-length S8 peptidase sequences (*n* = 54) were retrieved from UniProtKB (Supplementary Data Set S1). Mature protein regions were defined either according to curated UniProtKB processing annotations or, when unavailable, as the region extending from 30 residues upstream of the Pfam Peptidase_S8 domain (PF00082) to the C-terminus. Catalytic domains were defined as the PF00082 envelope flanked by 10 residues on each side and truncated at protein termini. Pfam domain boundaries were identified using hmmscan in HMMER v3.4 against the Pfam PF00082.28 HMM with the curated gathering threshold. Mature sequences were aligned using MAFFT v7.450 with the “--auto” option (Katoh and Standley, 2013). Maximum-likelihood phylogenetic analysis was performed using IQ-TREE 3.0.1 with the optimal substitution model (WAG+F + I + G4) selected by ModelFinder Plus. Branch support was assessed using 1,000 ultrafast bootstrap and 1,000 SH-aLRT replicates.

For detailed sequence comparisons, seven representative keratinases with experimentally characterized biochemical properties were analyzed together with Fervitase (Supplementary Table S1). Multiple sequence alignments were generated using Clustal Omega (EMBL-EBI) and visualized using ESPript 3.0 (Robert and Gouet, 2014). Biochemical properties of reference keratinases were compiled from published literature and the BacDive database (Schober et al., 2025).

### 2.2 Structural modeling and analysis

Experimentally determined protein structures were obtained from the Protein Data Bank (PDB) (Supplementary Table S1). Proteins lacking experimentally determined structures were modeled using AlphaFold3 via the AlphaFold Server based on their mature protein sequences, and the top-ranked model was selected. Unless otherwise noted, all predicted structures correspond to apo forms because no ligands or metal ions were included during modeling. Bound ligands and ions were removed from PDB-derived structures prior to structural comparison. For analysis of metal-coordinating residues, additional AlphaFold3 models were generated with different numbers of bound Ca^2+^ ions. The model containing three Ca^2+^ ions was selected because it provided geometrically plausible coordination at the predicted metal-binding sites and was used to identify putative coordinating residues. These sites were further validated against the FerB crystal structure (PDB: 9RK9).

Structural alignments were performed using the MatchMaker tool in UCSF ChimeraX (Pettersen et al., 2021). Peptide docking against Fervitase was conducted using HPEPDOCK 2.0 (Zhou et al., 2018) with the keratin-derived peptide sequence (“FSGRLTPPGTLH”) as the ligand. Top-ranked docking poses were subjected to local rigid-body relaxation, during which the receptor and peptide internal geometries were fixed and the peptide was translated by up to 2.5 Å to relieve interfacial clashes rather than by force-field minimization. Refined poses were filtered based on interface-contact quality, steric clashes, hydrogen-bond and salt-bridge content, and contact retention. The final docking model was selected by re-ranking the retained poses using the HPEPDOCK ITScore together with geometric penalties. Surface lipophilicity was calculated and visualized using the molecular lipophilicity potential (MLP) tool implemented in UCSF ChimeraX.

### 2.3 Cloning, mutagenesis, and expression of NA23_05775 and its variants

The NA23_05775 gene from *F. islandicum* AW-1, excluding residues 1–21, which correspond to the union of the native signal peptide regions predicted using SignalP 5.0 and 6.0 (Teufel et al., 2022; Armenteros et al., 2019), was amplified by PCR from genomic DNA isolated using a genomic DNA extraction kit (Qiagen, Hilden, Germany). The amplified PCR product was cloned into the pET-28a(+) vector (Novagen, San Diego, CA, USA) using the corresponding BamHI and XhoI restriction sites to generate pET-28a_Fervitase, encoding an N-terminal 6 × His-tagged recombinant protein.

The recombinant plasmid was transformed into *Escherichia coli* BL21(DE3). Transformants were cultured in Luria-Bertani (LB) medium supplemented with kanamycin (50 μg/mL) at 37 °C with shaking until OD_600_ reached 0.4–0.6. Protein expression was induced with 1 mM isopropyl-*β*-D-1-thiogalactopyranoside (IPTG), followed by overnight incubation at 37 °C. Cells were harvested by centrifugation (10,000 × g, 10 min, 4 °C) and stored at −20 °C until purification.

To investigate structure–function relationships, two site-directed alanine-substitution mutants and four truncation mutants targeting Fervitase-specific structural regions were generated (Supplementary Table S2). Mutagenesis PCR was performed using PrimeSTAR Max DNA Polymerase according to the manufacturer’s instructions. PCR products were treated with DpnI to remove template DNA and transformed into *E. coli* BL21 (DE3). All mutations were verified by DNA sequencing prior to protein expression and purification.

### 2.4 Purification of recombinant Fervitase

Cell pellets were resuspended in His-binding buffer (20 mM Tris–HCl, 500 mM NaCl, 5 mM imidazole; pH 7.9) and disrupted by ultrasonication on ice. After centrifugation (10,000 × g, 30 min, 4 °C), the supernatant was filtered through a 0.45-μm membrane and applied to a His-bind resin column (Novagen) pre-equilibrated with binding buffer. The column was sequentially washed with wash buffer A (20 mM Tris–HCl, 500 mM NaCl, 60 mM imidazole; pH 6.5) and wash buffer B (20 mM Tris–HCl, 500 mM NaCl, 60 mM imidazole; pH 7.9), and bound proteins were eluted using 250 mM imidazole. Active fractions were pooled, concentrated using a 30 kDa cutoff ultrafiltration device (Amicon Ultra, Millipore), and buffer-exchanged into 10 mM Tris–HCl (pH 7.9) containing 100 mM NaCl.

The oligomeric state of Fervitase was analyzed by size-exclusion chromatography (SEC) using a HiLoad 16/600 Superdex 200 pg column (GE Healthcare) equilibrated with 10 mM Tris–HCl (pH 7.9) and 100 mM NaCl. Molecular masses were estimated using calibration standards including thyroglobulin, ferritin, aldolase, conalbumin, ovalbumin, carbonic anhydrase, and ribonuclease A. Protein concentrations were determined using the bicinchoninic acid (BCA) assay with bovine serum albumin (BSA) as the standard. Protein purity and apparent molecular mass were analyzed by 10% SDS-PAGE.

### 2.5 Keratinase and protease activity assays

Keratinase activity was determined by quantifying free amino groups released during feather keratin hydrolysis using the ninhydrin colorimetric assay (Rosen, 1957). Unless otherwise noted, the standard reaction mixture (5 mL) contained 20 mM HEPES (pH 8.0, adjusted at 80 °C), 10 mM NaCl, 0.5% (w/v) native feather substrate, 10 mM dithiothreitol (DTT), 1 mM CaCl_2_, and 10 μg of purified Fervitase. Reactions were incubated at 80 °C for 1 h and terminated by adding 25 μL of 50% trichloroacetic acid (TCA) to 100 μL of reaction sample, followed by incubation on ice for 10 min. After centrifugation (12,000 × *g*, 10 min), 15 μL of supernatant was mixed with 75 μL of 3% ninhydrin solution and acetate–cyanide buffer (pH 5.2), heated at 100 °C for 15 min, cooled on ice, diluted with 50% (v/v) isopropanol, and measured at 570 nm. One unit (U) of keratinase activity was defined as the amount of enzyme that produced 1 nmol of free amino groups (arginine equivalent) per minute under the assay conditions.

Proteolytic activities toward soluble substrates were evaluated using casein and BSA. Reaction mixtures contained 0.2% (w/v) casein or 10 μM BSA in 20 mM Tris–HCl buffer (pH 8.0). Comparative assays were performed using Fervitase (80 °C), fervidolysin (72 °C), subtilisin E (60 °C), proteinase K (60 °C), and trypsin under their respective optimal reaction temperatures. Reactions containing trypsin, fervidolysin, or Fervitase additionally included 1 mM CaCl_2_. Following incubation for 30 min, reactions were terminated with 50% (w/v) TCA and centrifuged at 15,000 × *g* for 10 min at 4 °C. Proteolytic activity was quantified by measuring absorbance of the supernatant at 280 nm. One unit (U) of activity was defined as an increase of 0.01 absorbance units per minute. All experiments were performed in triplicate, and data are presented as mean ± standard deviation (SD).

### 2.6 Preparation of recombinant feather keratin substrates

Soluble recombinant keratin substrates were prepared as previously described (Jin et al., 2017). Briefly, *E. coli* cells expressing recombinant keratin were disrupted by ultrasonication in lysis buffer (50 mM NaH_2_PO_4_, 300 mM NaCl, 10 mM imidazole, 1 mM PMSF, pH 8.0). Inclusion body pellets were recovered by centrifugation (10,000 × *g*, 30 min) and solubilized in the same buffer containing 8 M urea for 1 h on ice. After centrifugation at 16,000 × *g* for 30 min, the supernatant was filtered through a 0.45-μm membrane and applied to a Ni-NTA agarose resin (Qiagen, Germany). Bound proteins were eluted with 250 mM imidazole, concentrated using centrifugal ultrafiltration devices (MWCO 3,000; Millipore), and refolded by stepwise dialysis against 50 mM Tris–HCl (pH 8.0) at 4 °C. Insoluble aggregates were removed by centrifugation, and soluble refolded keratin fractions were concentrated and stored at −80 °C until use.

### 2.7 Biochemical characterization and metal dependence of Fervitase

The effects of additives, temperature, pH, inhibitors, and divalent metal ions on Fervitase activity were evaluated under standard assay conditions unless otherwise indicated. Concentrations of CaCl_2_ (0–25 mM), NaCl (1–100 mM), and DTT (0–25 mM) were independently varied at 72 °C.

For temperature dependence, reactions containing 5 μg purified Fervitase were incubated at temperatures ranging from 50 to 100 °C for 1 h. The pH dependence of enzymatic activity was evaluated using overlapping 20 mM buffer systems including sodium acetate (pH 4.0–6.0), MES (pH 6.0–7.0), HEPES (pH 7.0–8.0), TAPS (pH 8.0–9.0), and borate (pH 9.0–10.0). All pH values were adjusted at 80 °C, and temperature-dependent ΔpKa/ΔT corrections were considered where appropriate.

To determine protease class, purified Fervitase (5 μg) was pre-incubated for 30 min at 50 °C with pepstatin A (10 μM), iodoacetamide (IAA; 1 mM), EDTA (1 mM), or phenylmethylsulfonyl fluoride (PMSF; 1 mM). Residual activity was subsequently measured under standard assay conditions at 80 °C.

For metal-dependence analysis, purified Fervitase was treated with 20 mM EDTA for 2 h at 50 °C and extensively dialyzed against 10 mM Tris–HCl (pH 8.0) at 4 °C to generate the apo enzyme. Metal contents of native and EDTA-treated enzymes were quantified by inductively coupled plasma mass spectrometry (ICP-MS) using an Agilent 7900 system. To assess metal activation, apo-Fervitase was pre-incubated for 30 min at 50 °C in the presence of 1 mM FeCl_2_, MgCl_2_·6H_2_O, NiSO_4_·6H_2_O, CaCl_2_·2H_2_O, CoCl_2_·6H_2_O, ZnCl_2_, MnCl_2_·4H_2_O, or CuSO_4_·2H_2_O prior to activity measurements under standard assay conditions.

### 2.8 LC–MS/MS analysis of Fervitase cleavage specificity

To investigate the substrate cleavage specificity of Fervitase, recombinant chromosome 2 feather keratin (Chr2; 300 μg) was digested under standard reaction conditions at 72 °C. For preparation of low-molecular-weight (LMW, <10 kDa) peptide fractions, reaction mixtures were subjected to ultrafiltration using a 10-kDa cutoff membrane (Amicon Ultra, Millipore). Filtrates were desalted using DK-Tip C18 columns, lyophilized, reconstituted in 0.1% formic acid (FA), and stored at 4 °C until analysis.

For in-solution digestion, Chr2 was solubilized in 6 M urea and 20 mM HEPES buffer (pH 8.0), reduced with 10 mM DTT at 56 °C for 30 min, and alkylated with 20 mM iodoacetamide (IAA) for 45 min in the dark at room temperature. After dilution to reduce the urea concentration below 1 M, purified Fervitase (10 μg) and 1 mM Ca^2+^ were added, and digestion was performed overnight at 72 °C. Peptides were subsequently desalted and processed as described above.

Peptide fractions were analyzed using an UltiMate 3000 RSLC nano system coupled to a Q-Exactive Orbitrap HF-X mass spectrometer (Thermo Fisher Scientific). Peptides were separated on Acclaim PepMap 100 C18 trap column (ID = 75 μm × 2 cm, 3 μm, 100 Å; Thermo Fisher Scientific) and eluted onto an analytical column (ID = 75 μm × 50 cm, packed with PepMap RSLC C18, 2 μm, 100 Å; Thermo Fisher Scientific) at a flow rate of 0.27 μL/min. Mobile phase A for LC separation was 0.1% FA in deionized water, while mobile phase B was 0.1% FA in acetonitrile (ACN). The chromatography gradient was programmed to begin with 5% solvent B for 5 min, followed by an increase to 10% B over the next 5 min. The proportion of solvent B was then ramped linearly to 35% over 65 min, increased to 50% B over 10 min, and further raised to 80% B in 1 min. The column was held at 80% B for an additional 18 min before being returned to 5% B in the final 1 min of the run. Tandem mass spectra were acquired in data-dependent acquisition mode over an m/z range of 300–1800. The spray voltage and capillary temperature were set to 2.5 kV and 250 °C, respectively, with normalized collision energy of 27% for MS/MS analysis.

Raw LC–MS/MS files were processed using MaxQuant v2.7.5.0 with unspecific digestion mode. Oxidation (M) and protein N-terminal acetylation were included as variable modifications, whereas carbamidomethylation (C) was set as a fixed modification. False discovery rates (FDRs) for peptide-spectrum matches and protein identification were both set to ≤ 1% (all other parameters were kept at default settings).

Cleavage preferences of Fervitase were visualized using sequence logos and the Chr2_FK4 peptide overlap frequency maps generated with the ggseqlogo and ggplot2 packages in R (Wagih, 2017). All scripts used for visualization are freely available at the GitHub repository.

### 2.9 Structural and thermal stability analyses

Circular dichroism (CD) spectroscopy was performed using a J-1500 CD spectrometer (JASCO, Tokyo, Japan). Protein samples were prepared in 10 mM Tris–HCl buffer (pH 7.9) containing 100 mM NaCl at a final concentration of 0.1 mg/mL. Spectra were recorded using a 1 cm path-length quartz cuvette with an internal volume of 0.35 mL over a wavelength range of 190–260 nm at room temperature. Buffer spectra were subtracted from all measurements, and spectra were averaged from three independent scans.

Thermal stability of Fervitase was further analyzed using a MicroCal PEAQ-DSC instrument (Malvern Instruments Ltd., Malvern, UK). Protein samples (1 mg/mL) prepared in 10 mM potassium phosphate (pH 7.9) containing 100 mM NaCl were scanned from 20 to 100 °C at a scan rate of 1 °C/min. Thermograms were baseline-corrected using corresponding buffer scans, and melting temperatures (T_m_) were determined using the manufacturer-provided analysis software. All measurements were performed in duplicate.

## 3 Results

### 3.1 Sequence, phylogenetic, and structural analyses identify Fervitase as a thermitase-like S8 protease

**Figure 1:**
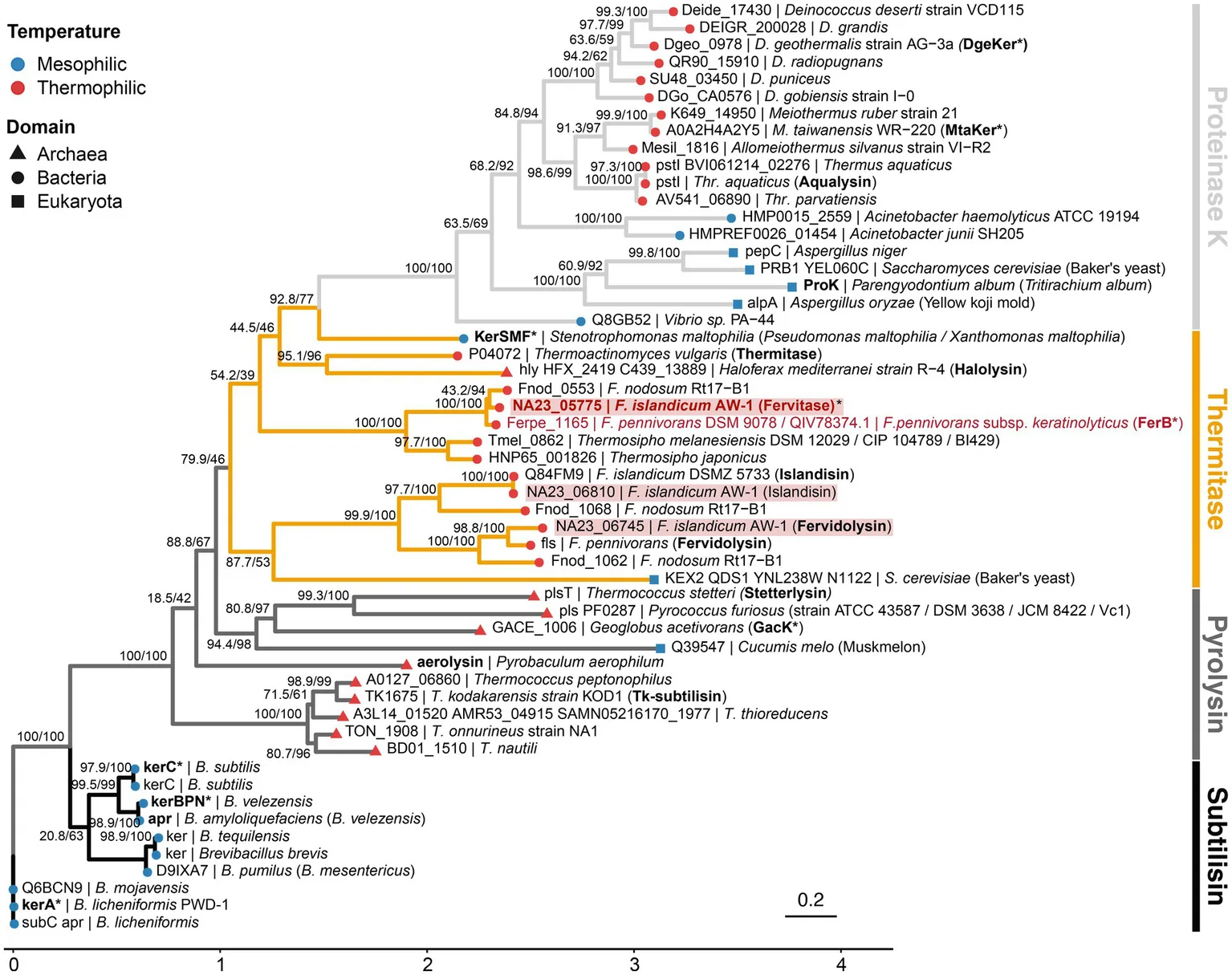
Evolutionary placement of Fervitase among thermitase-like S8 proteases. Maximum-likelihood phylogenetic tree of representative subtilisin-like S8 proteases constructed from catalytic-domain amino acid sequences using MAFFT alignment and IQ-TREE analysis. Branch support was evaluated by 1,000 SH-aLRT and 1,000 ultrafast bootstrap replicates. Fervitase from *F. islandicum* AW-1 clustered within the thermitase clade together with other thermophilic keratinolytic proteases, including fervidolysin and islandisin. Symbols indicate the taxonomic domain of origin (Archaea, Bacteria, or Eukaryota), whereas node colors indicate thermophilic or mesophilic origin. Previously characterized keratinases are marked with asterisks. The scale bar represents 0.2 amino acid substitutions per site. The sequences used for the alignment were listed in Supplementary Data Set S1.

Among the three S8 proteases encoded in *F. islandicum* AW-1, the compact protease encoded by NA23_05775 (designated Fervitase) remained previously uncharacterized. Unlike the previously characterized fervidolysin and islandisin, Fervitase showed marked transcriptional upregulation under nutrient-deficient feather-degrading conditions, strongly implicating its involvement in keratinolysis. Based on previous bioinformatic annotation (Kang et al., 2020), Fervitase was classified as a member of the peptidase S8 (subtilisin-like) family. To define its evolutionary relationship with known keratinases, a maximum-likelihood phylogenetic tree was constructed using the catalytic-domain sequences from 54 representative subtilisin-like proteases, including thermophilic and mesophilic keratinases (Supplementary Data Set S1) (Godde et al., 2005). The resulting phylogeny resolved four major clades corresponding to the thermitase, subtilisin, proteinase K, and pyrolysin lineages (Figure 1). Fervitase clustered unambiguously within the thermitase clade together with *Fervidobacterium*-derived proteases, including fervidolysin and islandisin, and was positioned adjacent to aerolysin from the hyperthermophilic archaeon *Pyrobaculum aerophilum*.

**Figure 2:**
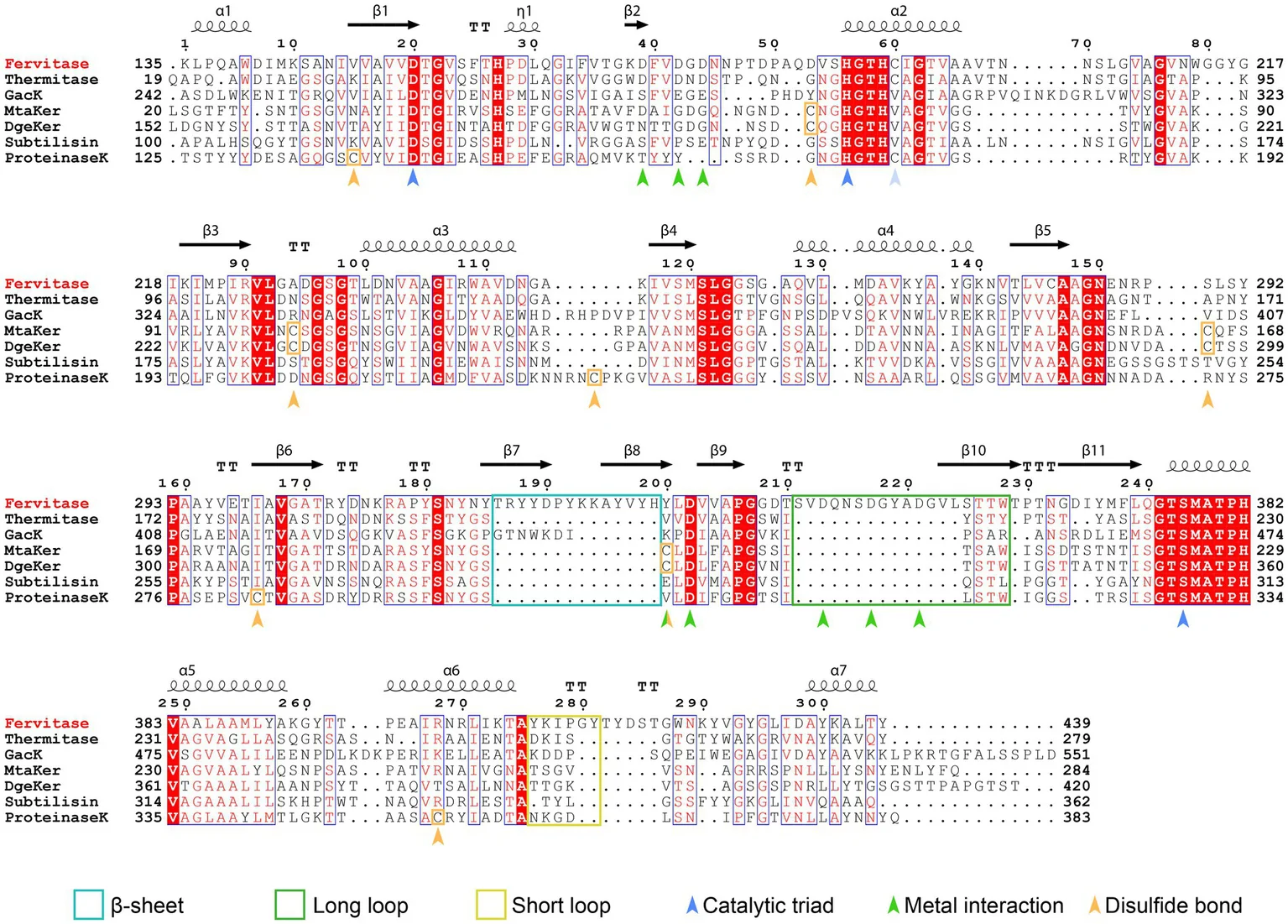
Structure-guided multiple sequence alignment of Fervitase and representative subtilisin-like proteases. The catalytic domain of Fervitase was aligned with representative members of the subtilisin superfamily, including thermitase, GacK, MtaKer, DgeKer, subtilisin, and Proteinase K. Secondary structural elements shown above the alignment correspond to the predicted structure of mature Fervitase, with *α*-helices represented as coils and *β*-strands as arrows. Conserved catalytic triad residues (Asp154-His190-Ser377) are indicated by blue arrowheads, whereas predicted metal-coordinating residues are indicated by green arrowheads. Conserved cysteine residues implicated in disulfide bond formation are marked with orange arrowheads. Fervitase-specific structural elements, including the extended β-sheet, long-loop, and short-loop regions, are highlighted by cyan, green, and yellow boxes, respectively. Conserved amino acid residues are highlighted in red.

**Figure 3:**
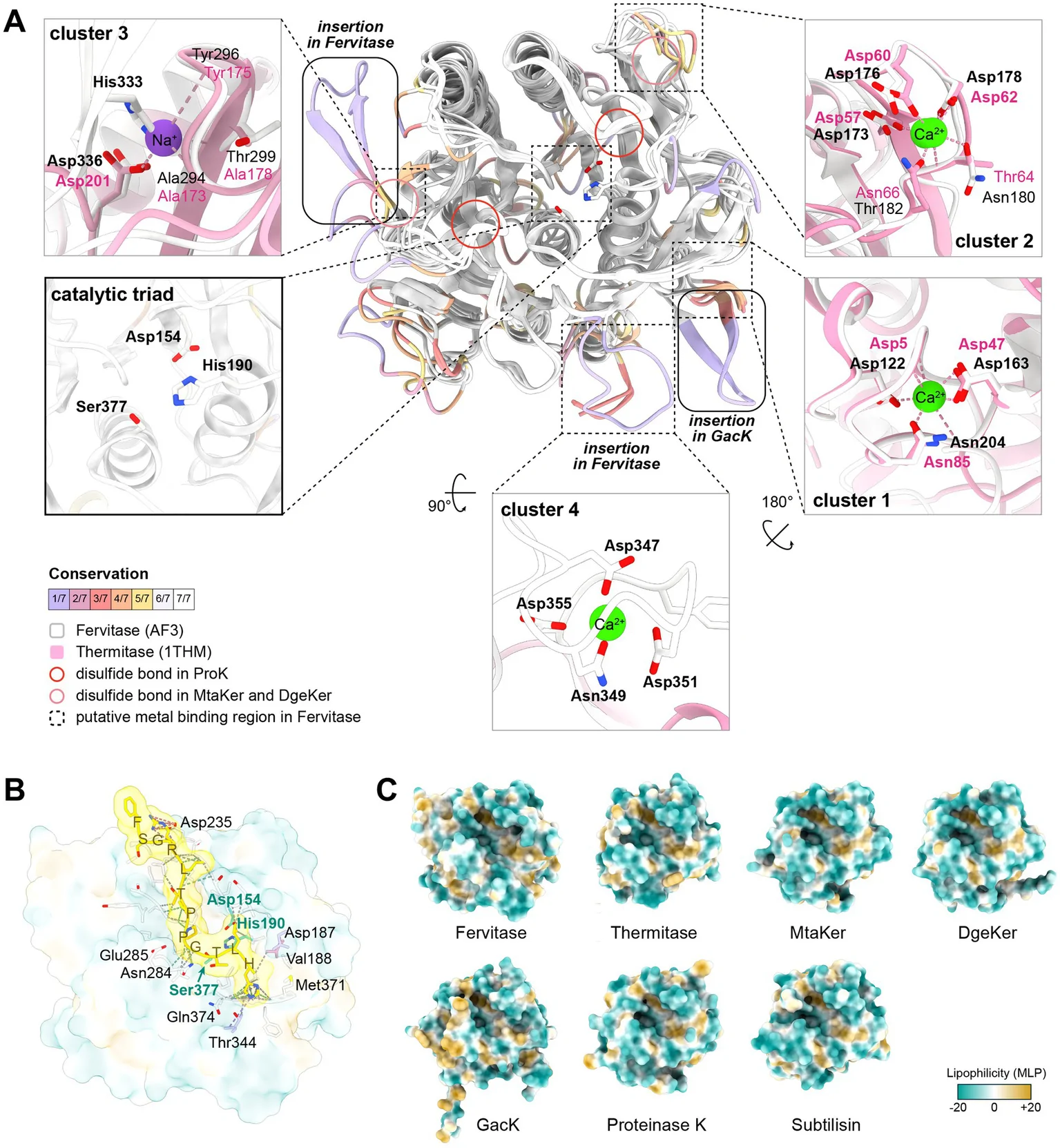
Structural features associated with thermophilic keratinolytic activity of Fervitase. **(A)** Structural superposition of Fervitase (Fvt) and representative subtilisin-like keratinases, including thermitase, MtaKer from *M. taiwanensis*, DgeKer from *D. geothermalis*, GacK from *G. acetivorans*, Proteinase K from *Tritirachium album*, and subtilisin from *B. subtilis*. Conserved catalytic cores are shown in gray, whereas Fervitase-specific insertions and extended structural elements are highlighted. Dashed boxes indicate putative metal-binding regions, and colored dotted circles indicate disulfide bond-forming residues identified in related keratinases. **(B)** Predicted peptide-binding mode of Fervitase generated using HPEPDOCK. A keratin-derived peptide substrate is shown in yellow within the substrate-binding cleft. Catalytic residues and predicted interacting residues are indicated. The model suggests an extended substrate-binding groove compatible with keratin-derived peptides. **(C)** Surface lipophilicity comparison of Fervitase and representative subtilisin-like proteases. Hydrophobicity was visualized using molecular lipophilicity potential (MLP) mapping in UCSF ChimeraX, where brown and cyan regions represent hydrophobic and hydrophilic surfaces, respectively.

Pairwise sequence comparisons with representative S8 proteases revealed moderate but significant conservation of the catalytic scaffold (Figure 2). Fervitase shared 41% sequence identity with thermitase, 37% with subtilisin, 33% with DgeKer, 31% with MtaKer and Proteinase K, and 27% with GacK. Multiple sequence alignment generated using ESPript 3.0 demonstrated conservation of the canonical Asp154-His190-Ser377 catalytic triad, confirming that Fervitase belongs to the subtilisin-like serine protease superfamily (Figure 2). Structural modeling using AlphaFold3 (Abramson et al., 2024) further supported this classification. Structural superimposition of Fervitase with representative S8 proteases revealed strong conservation of the catalytic core architecture (Figure 3A). The aligned structures exhibited root-mean-square deviation (RMSD) values ranging from 0.75 to 0.94 Å across 62.8—88.1% of aligned Cα residues (Supplementary Table S1), indicating substantial structural similarity despite modest sequence conservation.

The catalytic triad and overall active-site architecture of Fervitase closely resembled those of thermitase. Notably, cluster 1 (Asp122, Asp163, and Asn204; Fervitase numbering) and cluster 2 (Asp173, Asp176, and Asp178) were identified as putative metal-binding sites, each corresponding positionally to canonical calcium-binding motifs resolved in the thermitase crystal structure (Figure 3A). In addition, cluster 3 (His333 and Asp336) and cluster 4 (Asp347, Asn349, Asp351, and Asp355) were located within Fervitase-specific extended regions and were predicted to provide additional metal-coordination sites. Among these predictions, metal coordination at clusters 1 and 4 was consistent with the FerB crystal structure (PDB: 9RK9), whereas the remaining predicted sites were not occupied in the reported structure (Krüger et al., 2026), possibly reflecting calcium-dependent occupancy similar to that previously observed for thermitase (Gros et al., 1991). These structural features suggest enhanced metal coordination that may contribute to stabilization under extremely thermophilic conditions as seen in Tk-subtilisin (Uehara et al., 2012), Thermitase (Gros et al., 1991), and MtaKer (Wu et al., 2017).

Disulfide bonds were identified only in aerobic keratinases, including DgeKer, MtaKer, and Proteinase K, consistent with their established contribution to oxidative thermostability (Figure 2) (Tang et al., 2021; Wu et al., 2017; Betzel et al., 1988). In contrast, metal ion coordination motifs were broadly conserved among thermophilic subtilisin-like proteases (Figures 2, 3A). In addition, Fervitase contained uniquely extended loop regions and hydrophobic *β*-sheet insertions absent from most reference proteases, suggesting potential roles in substrate interaction, ion coordination, and/or thermal stabilization. Because thermitase activity is strictly dependent on Ca^2+^ (Gros et al., 1991; Teplyakov et al., 1990), these structural features suggested that Fervitase also functions as a Ca^2+^-dependent serine protease, which was subsequently confirmed biochemically.

Docking analysis further indicated that Fervitase possesses an active-site architecture compatible with extended keratin-derived peptides (Figure 3B). The modeled peptide adopted an elongated conformation along a broad catalytic groove, positioning the scissile peptide bond adjacent to the catalytic Asp154-His190-Ser377 triad while interacting with residues distributed across an extended substrate-binding surface. Consistent with this observation, surface lipophilicity mapping revealed a relatively continuous substrate-binding groove composed of intermixed hydrophobic and polar regions, whereas reference subtilisin-like proteases displayed more compact or discontinuous surfaces (Figure 3C). These structural features may facilitate efficient accommodation of unfolded keratin peptides during hydrolysis.

Collectively, these sequence, phylogenetic, and structural analyses suggest that Fervitase is a thermitase-like S8 serine protease possessing distinctive structural adaptations associated with thermophilic keratin degradation, including extended substrate-binding surfaces, unique loop insertions, and enhanced metal-coordination features.

### 3.2 Recombinant expression and biochemical optimization of Fervitase

The NA23_05775 gene encoding Fervitase was heterologously expressed in *E. coli* BL21 (DE3) as an N-terminal hexahistidine (6 × His)-tagged recombinant protein. Following induction, the soluble fraction was recovered after cell disruption and purified by Ni^2+^-affinity chromatography. A heat-denaturation step (50–60 °C), commonly used during purification of thermophilic enzymes (La et al., 2020), was intentionally omitted to minimize self-cleavage, a phenomenon frequently observed among S8 proteases (Wang et al., 2023).

**Figure 4:**
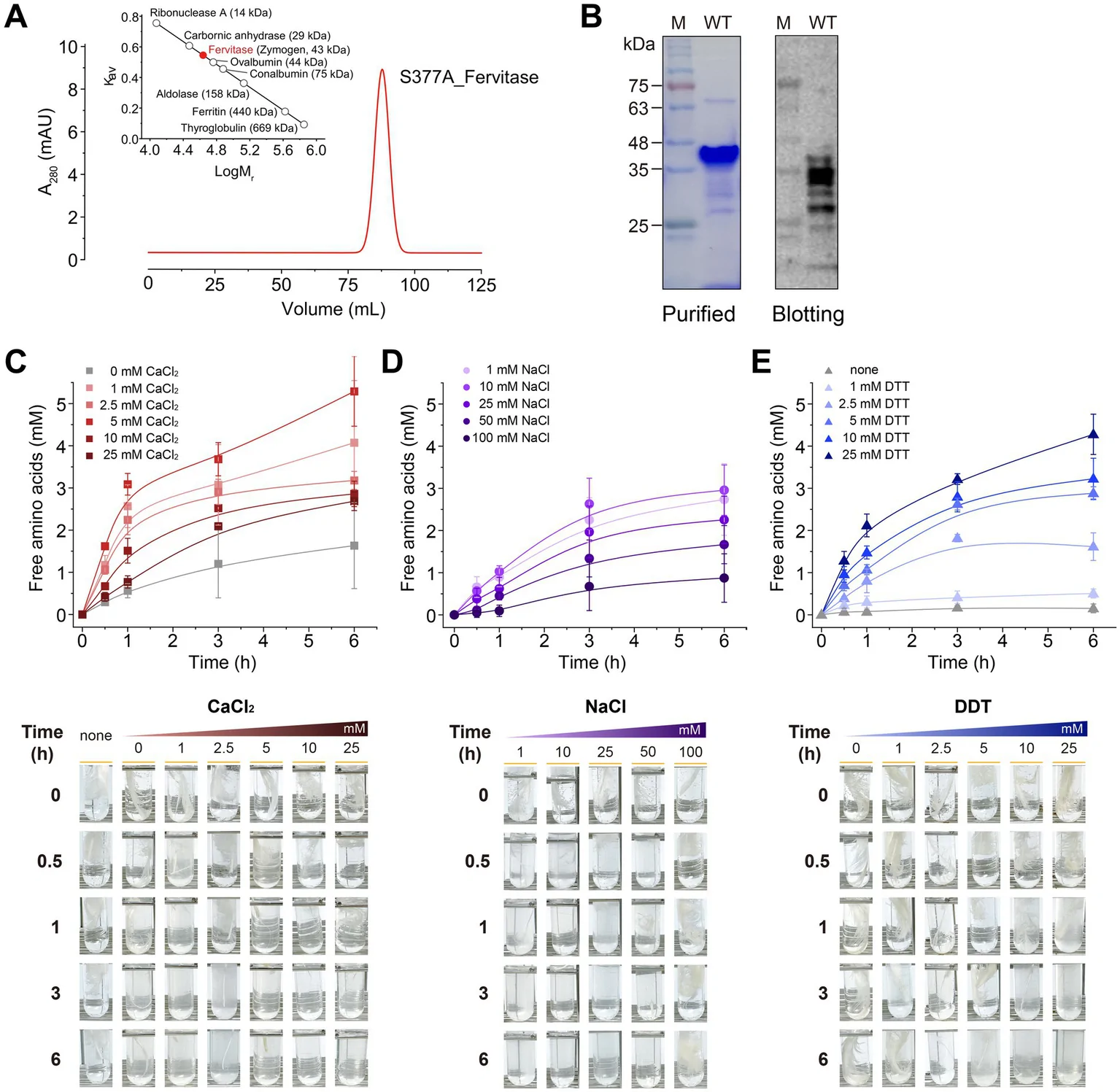
Purification of recombinant Fervitase and optimization of reaction conditions for keratin hydrolysis. **(A)** Size-exclusion chromatography (SEC) analysis of recombinant Fervitase S377A. The elution profile was compared with molecular weight standards, including thyroglobulin (669 kDa), ferritin (440 kDa), aldolase (158 kDa), conalbumin (75 kDa), ovalbumin (44 kDa), carbonic anhydrase (29 kDa), and ribonuclease A (14 kDa). The inset shows the calibration curve used for estimation of the apparent molecular mass of Fervitase S377A. **(B)** Purification and immunodetection of recombinant Fervitase. Purified protein was analyzed by 10% SDS–PAGE followed by Coomassie Brilliant Blue staining (left) and western blotting using an anti-His antibody (right). Lane M, molecular weight marker; lane WT, purified recombinant Fervitase. **(C–E)** Optimization of reaction conditions for keratin hydrolysis by Fervitase using native chicken feathers as substrate. Free amino acid release was quantified by the ninhydrin assay. Data are presented as mean ± SD (*n* = 3). Representative images of feather degradation are shown below each graph. **(C)** Effect of Ca^2+^ concentration (0–25 mM) on Fervitase activity**. (D)** Effect of NaCl concentration (1–100 mM NaCl) on Fervitase activity. Reactions were performed under otherwise standard assay conditions at 72 °C. **(E)** Effect of DTT concentration (0–25 mM) on Fervitase activity. Statistical significance was determined using one-way ANOVA followed by Tukey’s multiple comparisons test.

To determine the oligomeric state of Fervitase and verify its molecular mass, size-exclusion chromatography (SEC) was performed using a Superdex 200 pg column calibrated with standard proteins. To avoid autolysis-induced heterogeneity, the catalytically inactive Fervitase S377A mutant was used. Fervitase S377A eluted as a single symmetric peak corresponding to an apparent molecular mass of approximately 43.3 kDa, indicating that the enzyme exists predominantly as a monomer in solution and closely matches the calculated mass of the full-length zymogen (Figure 4A). SDS-PAGE analysis of catalytically active recombinant Fervitase revealed a major band at ~45.1 kDa, approximately 4 kDa smaller than the theoretical full-length mass (49.2 kDa) but still larger than the expected mature form following complete prodomain removal (Figure 4B). This observation suggests that recombinant Fervitase undergoes partial autocatalytic processing prior to complete maturation. Consistently, anti-His Western blotting detected multiple smaller immunoreactive fragments below the major band, indicating ongoing autoproteolytic cleavage and confirming that the purified enzyme retained catalytic activity in solution (Figure 4B).

To evaluate keratinolytic activity, preliminary assays were performed using 0.5% (w/v) native chicken feathers under aerobic conditions at 72 °C in the presence of 1 mM CaCl_2_, 1 mM NaCl, and 10 mM DTT. Under these conditions, Fervitase almost completely degraded feather substrates within 5 h, leaving only the central rachis structure intact (Figures 4C–E). This degradation rate exceeded that previously reported for the thermophilic keratinase MtaKer (Wu et al., 2017; Kluskens et al., 2002), prompting systematic optimization of reaction parameters. Because structural analysis identified multiple putative calcium-binding residues (Figures 2, 3A), the effect of Ca^2+^ supplementation was first examined (Figure 4C). Calcium strongly stimulated keratin hydrolysis, and even 1 mM Ca^2+^ markedly increased free amino acid release relative to the calcium-free control. In contrast, feather substrates incubated without enzyme remained visually intact throughout the assay period, confirming that degradation was enzyme-dependent. While 5 mM CaCl_2_ yielded the highest mean activity, no statistically significant differences were observed among 1, 2.5, and 5 mM CaCl_2_ after 6 h of incubation (*p* > 0.05). Therefore, 1 mM CaCl_2_ was selected for subsequent experiments because it supported robust hydrolysis while minimizing potential precipitation or inhibitory effects at higher ion concentrations.

The influence of ionic strength was next evaluated using NaCl concentrations ranging from 1 to 100 mM (Figure 4D). Fervitase activity peaked at 10 mM NaCl, whereas higher salt concentrations progressively reduced keratin hydrolysis, with substantial inhibition observed at 100 mM NaCl. This reduction likely reflects electrostatic shielding and/or partial protein aggregation under elevated ionic strength conditions (Wang et al., 2010; Timasheff, 2002). Accordingly, 10 mM NaCl was adopted as the standard reaction condition. Because keratin degradation requires reduction of intermolecular disulfide bonds, the effect of DTT concentrations was also investigated (Figure 4E). Keratin hydrolysis increased proportionally with DTT concentration and reached maximal activity at 25 mM DTT. In the absence of DTT, little to no detectable hydrolysis occurred, confirming that reductive cleavage of keratin disulfide bonds is essential for efficient degradation (Nam et al., 2002). Considering both catalytic efficiency and potential redox side effects, 10 mM DTT was selected as the standard reducing condition for subsequent assays.

### 3.3 Fervitase exhibits thermostable and metal-dependent keratinolytic activity

**Figure 5:**
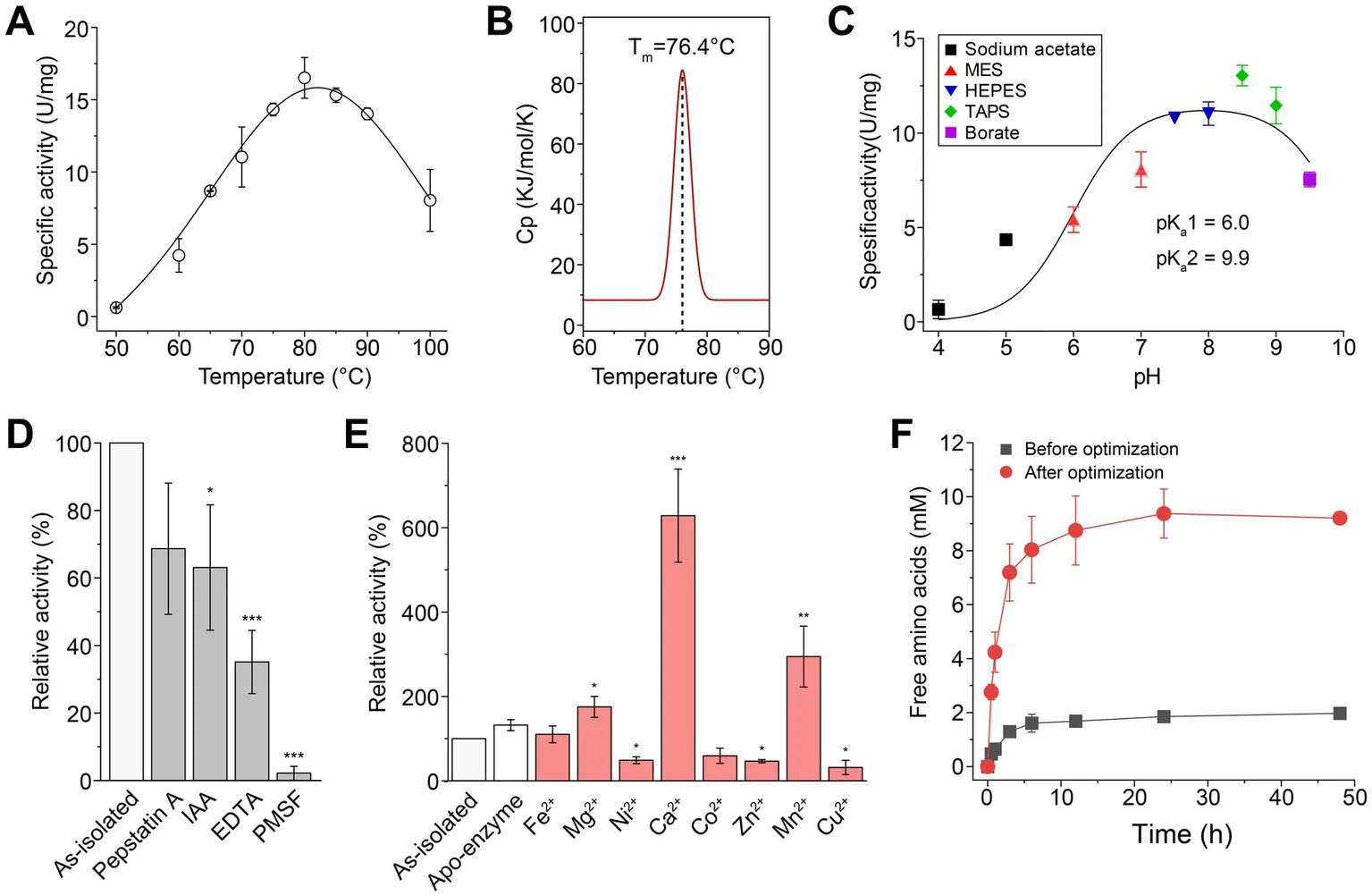
Biochemical and biophysical characterization of Fervitase. **(A)** Temperature dependence of Fervitase activity measured under standard assay conditions using native feather keratin as substrate. Reactions were incubated for 30 min at the indicated temperatures. **(B)** Differential scanning calorimetry (DSC) analysis of the catalytically inactive Fervitase S377A variant. The apparent melting temperature (T_m_) is indicated. **(C)** pH dependence of Fervitase activity measured using overlapping buffer systems: sodium acetate (pH 4.0–6.0), MES (pH 6.0–7.0), HEPES (pH 7.0–8.0), TAPS (pH 8.0–9.0), and borate (pH 9.0–10.0). Reactions were conducted for 60 min under otherwise standard assay conditions. **(D)** Effect of protease inhibitors on Fervitase activity. The enzyme was pre-incubated with pepstatin A, iodoacetamide (IAA), EDTA, or PMSF prior to activity measurement. **(E)** Effect of divalent metal ions on apo-Fervitase activity. EDTA-treated enzyme was reconstituted with 1 mM metal salts, pre-incubated at 50 °C for 30 min, and assayed for 60 min under standard conditions. Relative activity was normalized to the activity of the as-isolated enzyme. **(F)** Time-course analysis of free amino acid release from native chicken feathers under initial and optimized reaction conditions. All data are presented as mean ± SD from triplicate experiments. Statistical significance was assessed using one-way ANOVA followed by Tukey’s *post hoc* test (*p < 0.05, **p < 0.01, ***p < 0.001).

The temperature and pH dependence of Fervitase activity were examined under optimized standard assay conditions. Fervitase exhibited maximal activity at approximately 80 °C, which is notably higher than the optimal growth temperature (72 °C) of its native host, *F. islandicum* AW-1 (Nam et al., 2002) (Figure 5A). An Arrhenius analysis estimated the activation energy (E_a_) of the catalytic reaction to be 72.7 kJ/mol, consistent with efficient catalysis at elevated temperatures (data not shown). Thermal robustness was further confirmed by differential scanning calorimetry (DSC) analysis of the catalytically inactive S377A zymogen mutant, which showed a melting temperature (T_m_) of 76.4 °C (Figure 5B). The close agreement between T_m_ and the temperature optimum indicates that the catalytic scaffold remains structurally stable near its maximal operating temperature. Notably, substantial catalytic activity was retained even at 90 °C.

The effect of pH was evaluated using overlapping buffer systems spanning pH 4.0–10.0. Fervitase exhibited maximal activity at pH 8.5 in TAPS buffer (Figure 5C), consistent with the alkaline preference characteristic of subtilisin-like proteases. However, because TAPS can interfere with divalent cation-dependent enzymes through weak metal-chelating effects (Ferreira et al., 2015), HEPES buffer at pH 8.0 was selected for subsequent assays. Bell-shaped fitting of the pH profile yielded apparent pK_a_ values of 6.0 and 9.9, suggesting that ionizable catalytic residues contribute to activity across the alkaline range. Together, these results indicate that Fervitase is adapted for catalysis under thermo-alkaline conditions.

To experimentally validate the predicted enzyme class of Fervitase, inhibitor-sensitivity assays were performed using pepstatin A, iodoacetamide (IAA), EDTA, and PMSF (Figure 5D). Neither pepstatin A nor IAA significantly affected enzymatic activity, indicating that Fervitase does not rely on aspartic acid- or cysteine-dependent catalysis. In contrast, EDTA partially reduced activity, suggesting a requirement for metal ion-mediated stabilization. Most notably, PMSF treatment almost completely abolished activity (2.2% residual activity), providing direct biochemical evidence that Fervitase functions as a serine protease.

Because structural analyses predicted multiple Ca^2+^-binding residues within the catalytic scaffold, the contribution of divalent metal ions was further investigated (Zeng et al., 2014). Apo-enzyme was prepared by EDTA treatment followed by extensive dialysis, and metal contents were quantified by ICP-MS analysis. The as-isolated enzyme contained detectable levels of Mg^2+^ (0.11), Ca^2+^ (0.09), Mn^2+^ (0.007), Fe^2+^ (0.13), Co^2+^ (0.04), Ni^2+^ (0.32), Cu^2+^ (not detected), and Zn^2+^ (0.24) per enzyme subunit, whereas most bound metals were substantially depleted following EDTA treatment, confirming effective chelation. Interestingly, the apo-enzyme retained activity comparable to that of the as-isolated preparation, suggesting that the purified native enzyme was not fully saturated with activating Ca^2+^ ions. Consistent with this interpretation, supplementation with 1 mM Ca^2+^ increased activity to approximately 629% relative to the untreated enzyme (Figure 5E). Mn^2+^ also strongly stimulated activity (~295%), whereas Ni^2+^, Zn^2+^, and Cu^2+^ markedly inhibited catalysis. These findings indicate that Fervitase is a Ca^2+^-activated thermophilic subtilisin-like protease whose catalytic efficiency and structural stability are strongly enhanced by divalent cation coordination.

To assess the cumulative effect of optimized reaction parameters, final assay conditions were established using 1 mM CaCl_2_, 10 mM NaCl, and 10 mM DTT. Under these conditions, feather hydrolysis efficiency increased markedly relative to the initial assay conditions (Figure 5F). The concentration of released free amino acids increased approximately 4.8-fold, from 1.97 mM to 9.38 mM, whereas the apparent half-time (t_1/2_) for product formation decreased substantially, indicating accelerated substrate turnover and enhanced catalytic efficiency. Correspondingly, the initial reaction rate (V_0_) increased approximately 5.9-fold under optimized conditions.

Collectively, these findings establish Fervitase as a highly thermostable and Ca^2+^-activated serine protease that retains robust catalytic activity under thermo-alkaline conditions, highlighting its potential as a biocatalyst for high-temperature feather valorization.

### 3.4 Fervitase selectively targets native feather keratin

To investigate the substrate specificity of Fervitase, its proteolytic activity was compared with those of representative serine proteases, including proteinase K, subtilisin E, and trypsin, as well as the extremely thermophilic subtilisin-like protease fervidolysin. Proteinase K, subtilisin E, and trypsin efficiently hydrolyzed soluble protein substrates such as casein and bovine serum albumin (BSA), exhibiting activities ranging from 4.8 to 222 U/mg (Table 1). In contrast, neither Fervitase nor fervidolysin showed detectable activity toward these soluble substrates. However, when native chicken feathers were used as the substrate, Fervitase exhibited strong keratinolytic activity (38 ± 3.9 U/mg), whereas trypsin, subtilisin E, and fervidolysin showed little or no detectable degradation under the same conditions. Proteinase K retained measurable feather-degrading activity (5.2 ± 0.4 U/mg), although its activity remained substantially lower than that of Fervitase. Fervidolysin failed to degrade any tested substrate, likely due to incomplete prodomain maturation and insufficient catalytic activation.

**Table 1:** Comparison of proteolytic activities of representative serine proteases toward soluble proteins and native feather keratin.

Substrate	Protease (Unit/mg)				
	Proteinase K	Subtilisin E	Trypsin	Fervidolysin	Fervitase
Casein	8.1 ± 0.4	157 ± 24	222 ± 53	n.d.*	n.d.
BSA	4.8 ± 2.8	57 ± 20	139 ± 42	n.d.	n.d.
Native feather	5.2 ± 0.4	0.9 ± 0.2	n.d.	n.d.	38 ± 3.9

^*^ n.d. = not detected.

Notably, Fervitase selectively degraded the highly recalcitrant feather keratin while exhibiting negligible activity toward soluble proteins such as casein and BSA. This unusual substrate preference suggests that Fervitase recognizes structural features unique to native keratin, including its *β*-sheet–rich organization, high cystine content, and densely cross-linked architecture. These characteristics differ fundamentally from the flexible and solvent-accessible conformations of globular or intrinsically disordered proteins such as BSA and casein. Thus, the inability of Fervitase to hydrolyze soluble proteins likely reflects an evolutionary specialization toward compact keratin matrices rather than generalized proteolysis. Consistent with this interpretation, structural analyses revealed exposed hydrophobic regions surrounding the substrate-binding cleft together with several Fervitase-specific structural insertions (Figures 2, 3). Such hydrophobic surfaces may facilitate adsorption onto feather keratin fibers and enhance substrate recognition within insoluble keratin matrices.

**Figure 6:**
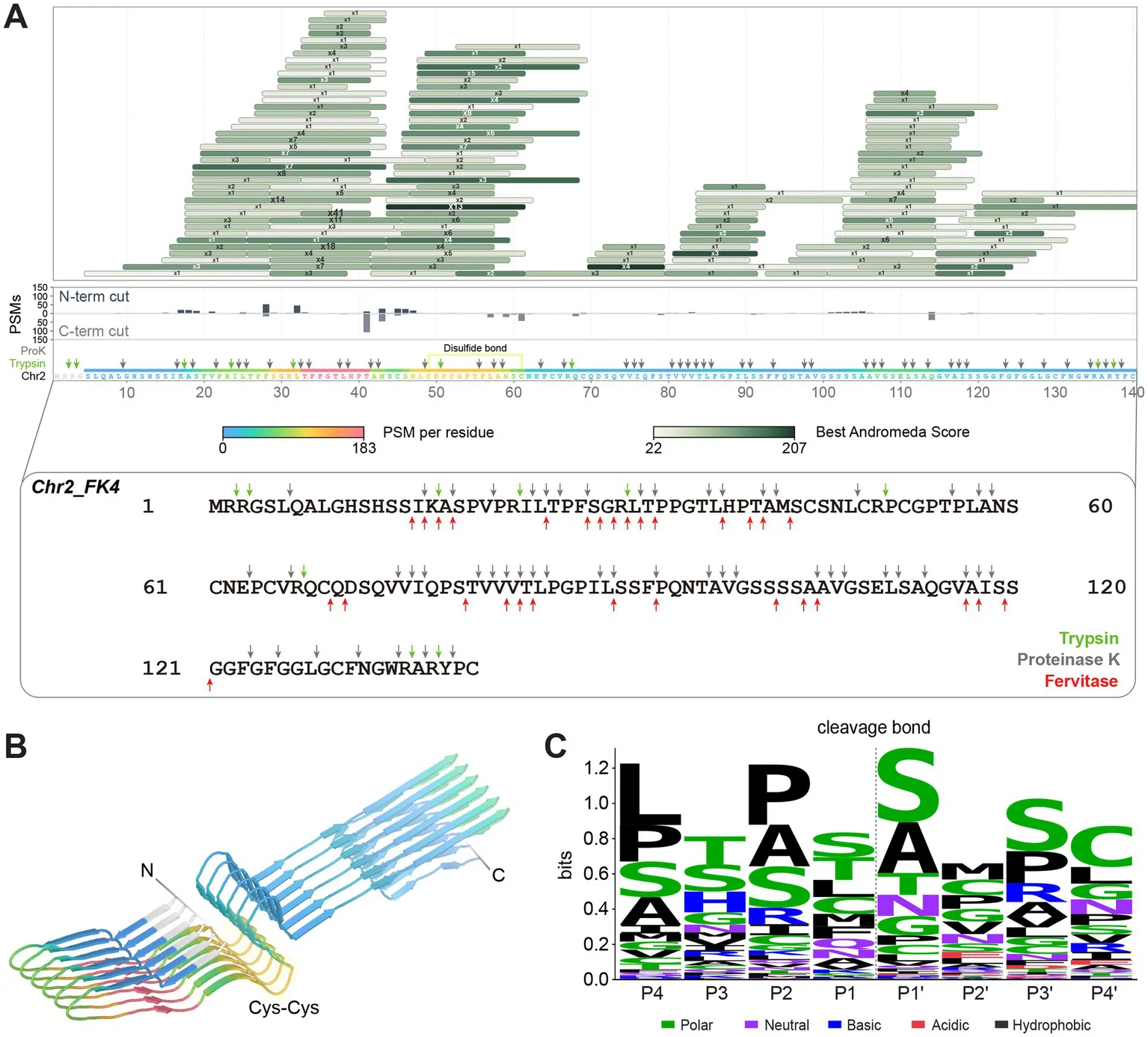
LC–MS/MS-based characterization of Fervitase cleavage specificity on recombinant feather keratin (Chr2). **(A)** Peptide coverage and cleavage-site mapping of Fervitase-digested Chr2 keratin. The upper panel shows LC–MS/MS-derived peptide overlap frequencies across the Chr2 sequence, with peptide-spectrum match (PSM) density and Andromeda scores indicated by color intensity. The middle panel summarizes predicted or experimentally identified cleavage sites generated by trypsin, proteinase K, and Fervitase. The lower panel shows residue-level cleavage-site mapping along the Chr2 sequence (residues 1–140), highlighting preferential cleavage regions and the predicted disulfide bond-forming cysteine residues. **(B)** Predicted structural model of recombinant Chr2 feather keratin. The β-sheet-rich architecture characteristic of feather keratin is shown, with the predicted disulfide bond indicated. **(C)** Sequence logo representation of amino acid frequencies surrounding Fervitase cleavage sites derived from LC–MS/MS analysis. P1 denotes the residue immediately preceding the cleavage site, whereas P1′ denotes the residue immediately following. Amino acids are colored according to physicochemical properties: hydrophobic (black), polar (green), neutral (purple), basic (blue), and acidic (red).

To further define cleavage specificity, LC–MS/MS analysis was performed using recombinant *Gallus* chromosome 2 (Chr2) feather keratin as the substrate (Figure 6). Peptide-spectrum match (PSM)-based peptide lists and cleavage-site information are summarized in Supplementary Table S3. Peptide overlap mapping demonstrated that Fervitase preferentially cleaved specific regions of Chr2 rather than indiscriminately hydrolyzing the entire sequence (Figure 6A). In particular, the peptide TPPGTLHPT was detected at markedly higher abundance than other fragments, and N-terminal mapping revealed enriched cleavage sites surrounding Ser29 and Thr33. These observations suggest that Fervitase preferentially targets relatively flexible regions enriched in small, non-bulky residues within the otherwise rigid keratin scaffold (Figures 6A,B).

Comparison of cleavage patterns among trypsin, proteinase K, and Fervitase revealed that the specificity of Fervitase more closely resembled that of proteinase K than that of trypsin (Figure 6A). Unlike trypsin, which preferentially cleaves after basic residues (Jin et al., 2017), Fervitase displayed relatively broad cleavage permissiveness without strict dependence on Lys or Arg residues, consistent with the catalytic behavior of subtilisin-family proteases. Sequence logo analysis further demonstrated preferential accommodation of Ser, Thr, Leu, and Cys at the P1 position and Ser, Ala, and Thr at the P1′ position (Figure 6C). These preferences partially overlap with those reported for other S8 serine proteases, including proteinase K, subtilisin E, and thermitase, but differ substantially from the strict basic-residue preference characteristic of trypsin-like proteases (Bromme and Kleine, 1984; Bromme et al., 1986; Tang et al., 2019; Schilling et al., 2018).

Importantly, the cleavage specificity of Fervitase could not be explained solely by P1/P1′ residue preference. Instead, efficient feather degradation likely reflects a combination of broad catalytic permissiveness and structural adaptation for interaction with insoluble keratin matrices. In particular, the exposed hydrophobic surface regions and Fervitase-specific structural elements identified above may facilitate stable interactions with the *β*-sheet-rich and cystine-crosslinked feather architecture, thereby enabling efficient keratin degradation under thermo-alkaline conditions.

### 3.5 Unique structural elements govern Fervitase maturation and keratinolytic activity

**Figure 7:**
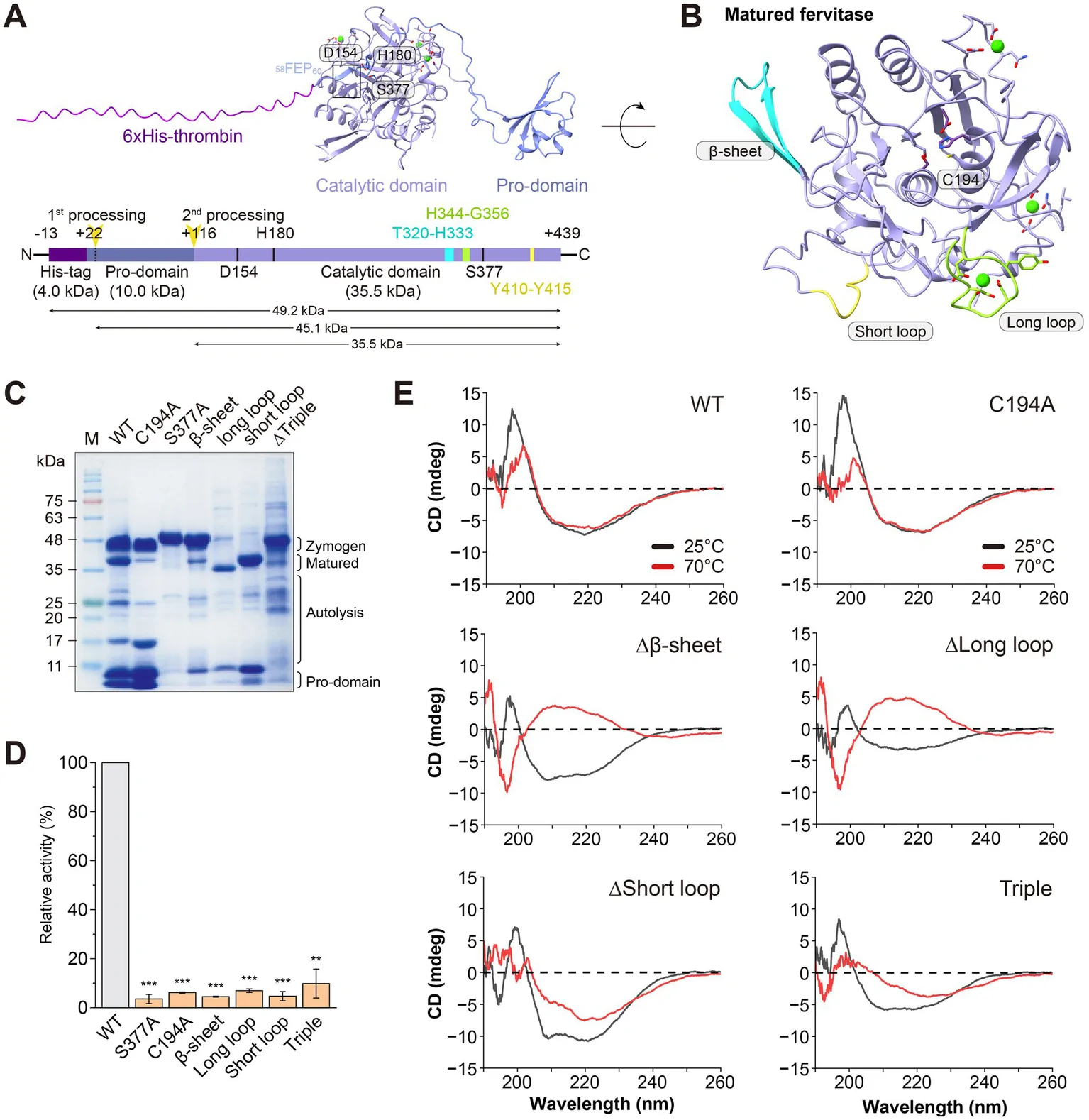
Structural determinants governing Fervitase maturation, thermostability, and keratinolytic activity. **(A)** Schematic representation of recombinant Fervitase domain organization and proposed maturation process. The N-terminal 6 × His-thrombin tag, pro-domain, catalytic domain, catalytic residues, and engineered structural regions are indicated. Predicted sequential autoprocessing events generating the mature enzyme are shown together with the corresponding molecular masses. **(B)** Structural model of mature Fervitase highlighting engineered structural elements. The β-sheet region, long loop, and short loop targeted for truncation mutagenesis are colored cyan, green, and yellow, respectively. Conserved residues associated with catalysis and structural stabilization are indicated. **(C)** SDS–PAGE analysis of wild-type Fervitase and engineered variants. Lane M, molecular weight markers; lane WT, wild-type Fervitase; lane C194A, catalytic-cavity cysteine mutant; lane S377A, catalytic serine mutant; lane Δβ-sheet, β-sheet truncation mutant; lane ΔLong loop, long-loop truncation mutant; lane ΔShort loop, short-loop truncation mutant; lane Triple, combined truncation mutant. Bands corresponding to zymogen, mature enzyme, autoproteolytic products, and released pro-domain are indicated. **(D)** Relative feather-degrading activities of Fervitase and engineered variants measured under standard assay conditions at 90 °C for 60 min. Activities were normalized to wild-type Fervitase. Statistical significance was assessed using one-way ANOVA followed by Tukey’s post hoc test (**p < 0.01, ***p < 0.001 versus WT). **(E)** Circular dichroism (CD) spectra of wild-type Fervitase and mutant variants measured at 25 °C and 70 °C. Thermal unfolding profiles reveal the contribution of Fervitase-specific structural elements to conformational stability at elevated temperatures.

To investigate the structural basis underlying the unique keratinolytic activity of Fervitase, we focused on structural differences between Fervitase and thermitase, a closely related thermophilic subtilisin-like protease that lacks feather-degrading activity (Figure 3A). Comparative structural analyses revealed that thermitase possesses shorter substrate-accommodating loops and a more compact β-sheet region, features that may restrict access to bulky keratin substrates. Based on these observations, four structure-guided deletion mutants targeting Fervitase-specific regions were generated: Δβ-sheet^T320–H333^, Δlong-loop^H345–G356^, Δshort-loop^Y410–Y415^, and a triple mutant combining all three deletions (Figures 7A,B). Notably, the long- and short-loop regions encompass a predicted Ca^2+^-binding region potentially involved in structural stabilization under thermophilic conditions.

In parallel, structural inspection of the catalytic cavity suggested an additional redox-sensitive feature. During preliminary assays, Fervitase activity was markedly reduced under strictly anaerobic conditions despite the requirement for reducing conditions during keratin degradation, suggesting that redox chemistry may influence not only substrate reduction but also enzyme catalysis itself. Structural modeling revealed that Cys194 is positioned within the catalytic cavity at distances from the catalytic His and Ser residues resembling the geometry of the canonical Asp-His-Ser catalytic triad. To evaluate its functional contribution, Cys194 was substituted with alanine (C194A). In addition, the catalytic serine residue (Ser377) was replaced with alanine to generate a catalytically inactive control mutant (S377A). In total, six Fervitase variants were generated for structural and functional characterization.

All variants were successfully expressed and purified, and their maturation states were analyzed by SDS–PAGE (Figure 7C). Wild-type (WT) Fervitase migrated predominantly as two major bands at approximately 45.1 and 35.5 kDa, whereas the catalytically inactive S377A mutant largely retained the full-length precursor form. Given the calculated molecular mass of the recombinant zymogen (49.2 kDa), the 45.1 kDa species likely corresponds to removal of the N-terminal His-tag and adjacent residues, while the 35.5 kDa band reflects additional prodomain excision. Because these processing events were abolished in the S377A mutant, both cleavage steps are interpreted as autocatalytic maturation events mediated by the intrinsic catalytic activity of Fervitase.

The keratinolytic activities of all mutants were subsequently evaluated using native feather substrates, and their structural integrity was analyzed by circular dichroism (CD) spectroscopy (Figures 7D,E). All mutants retained less than 10% of WT feather-degrading activity, demonstrating that each targeted structural region is essential for efficient keratinolysis. However, the mechanisms underlying activity loss differed substantially among mutants and could be classified into five functional failure modes: (i) catalytic inactivation (S377A), (ii) impaired autoprocessing despite preserved secondary structure (C194A), (iii) combined maturation defect and thermal destabilization (Δ*β*-sheet), (iv) thermal collapse despite apparent maturation (Δlong-loop and triple mutant), and (v) preserved maturation and structural stability but loss of catalytic function (Δshort-loop).

CD spectra collected at room temperature demonstrated that all mutants retained substantial secondary structure prior to heat treatment, indicating that activity loss was not simply caused by global misfolding. As expected, S377A completely lost catalytic activity due to disruption of the catalytic serine residue. In contrast, the C194A mutant retained stable secondary structure but failed to undergo efficient autocatalytic maturation, suggesting that Cys194 contributes indirectly to active-site organization and/or enzyme activation. The Δβ-sheet mutant exhibited defective maturation together with pronounced thermal precipitation at 70 °C, indicating that this β-sheet region contributes to both structural stabilization and efficient prodomain processing. Similarly, the Δlong-loop and triple mutants generated mature-sized protein bands on SDS–PAGE but underwent severe thermal destabilization during high-temperature incubation, demonstrating that these extended loop regions are essential for maintaining structural integrity under thermophilic conditions. Interestingly, the Δshort-loop mutant underwent apparently normal maturation and remained soluble at elevated temperatures yet retained almost no keratinolytic activity, suggesting that this region contributes to substrate positioning, or active-site dynamics rather than overall fold stability.

Collectively, these results demonstrate that multiple Fervitase-specific structural elements cooperatively govern enzyme maturation, catalytic activity, and thermostability. Rather than functioning independently, the *β*-sheet region, extended loops, catalytic residues, and redox-sensitive structural components together form an integrated thermophilic keratinolytic architecture required for efficient degradation under thermo-alkaline conditions.

## 4 Discussion

*Fervidobacterium islandicum* AW-1 is a well-known thermophilic FDB capable of decomposing intact chicken feathers within 48 h at 72 °C (Lee et al., 2015). Despite its extensive use as a model organism for thermophilic keratin degradation, the principal protease responsible for this activity has remained unresolved. Previous studies largely focused on fervidolysin, the most abundantly expressed S8 protease identified in transcriptomic analyses of *F. islandicum* AW-1 (Sung et al., 2026). However, although less abundant overall, Fervitase was strongly induced during active feather degradation, showing expression dynamics more consistent with a direct role in keratinolysis (Kang et al., 2020). Phylogenetic analysis placed all *Fervidobacterium* S8 proteases within the thermitase clade, indicating conservation of a common thermophilic catalytic scaffold and stabilization strategy (Figure 1). Unlike the compact architecture of Fervitase, fervidolysin and islandisin contain additional kexin-like accessory domains that may contribute to substrate association (Bhagya Jyothi and Dhanasingh, 2025; Kluskens et al., 2002). Among these enzymes, islandisin has been shown to become catalytically active following appropriate processing (Godde et al., 2005), suggesting that productive maturation, rather than overall fold conservation alone, is a critical determinant of functional activity among *Fervidobacterium* subtilisins.

Structural analysis revealed that Fervitase contains several distinctive thermophilic adaptations superimposed on the conserved thermitase-like catalytic core. In particular, the enzyme possesses an expanded acidic cluster composed of multiple Asp residues surrounding the putative metal-binding region (Figures 2, 3A). Similar metal-coordination motifs were variably distributed among thermophilic subtilisin-like proteases, including both conserved calcium-binding sites and species-specific extensions located within enlarged surface loops. Deletion of the *β*-sheet and loop regions containing these motifs caused severe thermal destabilization and/or loss of feather-degrading activity, demonstrating that these auxiliary structural elements are essential for maintaining catalytic competence under elevated-temperature conditions (Figure 7).

The apparent T_m_ of the Fervitase zymogen (~76 °C) closely matched the optimal catalytic temperature range, although substantial enzymatic activity persisted at 80 °C (Figures 5A,B). This discrepancy suggests that the mature enzyme may possess greater thermostability than the precursor form. Direct characterization of the mature enzyme was not feasible because the WT enzyme underwent rapid autolysis, whereas prodomain-deleted constructs failed to express stably. Future structural and calorimetric studies using properly matured preparations will therefore be required to resolve the thermal properties of the active catalytic state.

A defining feature of Fervitase was its pronounced preference for insoluble feather keratin over soluble protein substrates. Whereas proteinase K, subtilisin E, and trypsin efficiently hydrolyzed soluble proteins such as casein and BSA, Fervitase showed little or no detectable activity toward these substrates while exhibiting strong keratinolytic activity toward native feathers (Table 1). This selective substrate profile indicates that Fervitase is specialized for degradation of rigid, insoluble keratin matrices rather than broad-spectrum proteolysis.

Feather keratin differs fundamentally from soluble proteins because it contains densely packed *β*-sheet structures stabilized by extensive hydrophobic interactions and intermolecular disulfide cross-links. Structural comparisons identified several Fervitase-specific surface features, including extended loop regions, hydrophobic *β*-sheet elements, and exposed hydrophobic patches surrounding the substrate-binding cleft (Figure 3C). These features likely facilitate physical association with the hydrophobic keratin surfaces and help position partially unfolded substrate regions near the catalytic center. Consistent with this interpretation, the Δshort-loop mutant retained normal maturation and thermal stability yet almost completely lost feather-degrading activity, suggesting that this region contributes directly to substrate interaction rather than global structural stability (Figure 7).

LC–MS/MS-based cleavage-site mapping further demonstrated that Fervitase preferentially cleaves flexible regions of recombinant keratin rather than rigid β-sheet cores (Figure 6). Cleavage sites were enriched in small residues such as Ser, Thr, and Ala at the P1 position, consistent with the relatively constrained S1 pocket predicted by structural modeling. However, this sequence preference alone could not fully explain the strong keratin selectivity of Fervitase because similar residue preferences are shared among several other S8 proteases. Instead, the overall hydrophobic substrate-binding environment together with peripheral structural elements likely cooperates to enable productive recognition of insoluble feather keratin.

Another notable feature of Fervitase was its unusual maturation mechanism. Like many subtilisin-family proteases, Fervitase is synthesized as an inactive precursor containing an inhibitory prodomain that suppresses catalytic activity until proteolytic maturation occurs. The enzyme underwent two sequential autoprocessing events, yielding intermediate and mature forms with apparent molecular masses of approximately 45.1 and 35.5 kDa, respectively, both of which were abolished in the catalytically inactive S377A mutant (Figure 7). These results demonstrate that maturation depends on intrinsic autoproteolytic activity. Consistent with this interpretation, the FerB crystal structure, in which the prodomain was crystallized together with the mature enzyme, appears to represent a processing intermediate (Krüger et al., 2026). Structural inspection revealed that a short β-sheet element is positioned adjacent to the first cleavage site and near the catalytic center (Figures 7A–C). Similar transient β-sheet structures have been reported in several subtilisin-like proteases and may function as structurally accessible regions that facilitate autoproteolytic activation, suggesting that this element contributes to the maturation process (Tanaka et al., 2007). In addition, Cys194 occupied an unusual position within the catalytic cavity. This residue is typically replaced by valine in other S8 proteases, yet substitution of Cys194 with alanine severely impaired autoprocessing without substantially altering overall secondary structure (Figures 7C–E). Together with the marked reduction of activity under strictly anaerobic conditions, these findings suggest that Cys194 contributes to a redox-sensitive active-site configuration required for productive maturation and catalysis.

Collectively, our results demonstrate that Fervitase combines a conserved thermitase-like catalytic scaffold with peripheral structural adaptations specialized for keratin degradation under extremely thermophilic conditions. These adaptations include expanded metal-binding regions, hydrophobic substrate-interacting surfaces, and structural elements governing maturation and thermal stability. Such features likely enable efficient degradation of highly recalcitrant feather keratin while maintaining catalytic activity at elevated temperatures. Given the enormous quantities of keratin-rich poultry waste generated globally, Fervitase represents a promising candidate for high-temperature keratin bioconversion and sustainable protein valorization (Kim et al., 2026). Its strong keratin selectivity, thermostability, and structurally defined catalytic architecture also provide a valuable framework for future engineering of customized thermophilic biocatalysts for circular bioeconomy applications.

## 5 Conclusion

In this study, we identified and characterized Fervitase, a previously uncharacterized thermophilic feather-degrading S8 serine peptidase from *F. islandicum* AW-1. Phylogenetic and structural analyses demonstrated that Fervitase belongs to the thermitase subgroup of subtilisin-like proteases while possessing unique peripheral structural adaptations associated with keratin degradation and thermophilic stability. The enzyme exhibited strong keratinolytic activity toward native chicken feathers while showing minimal activity toward soluble protein substrates, indicating a high degree of substrate specialization.

Biochemical characterization demonstrated that Fervitase functions optimally under thermo-alkaline conditions and requires both Ca^2+^ and reducing conditions for efficient keratin degradation. Optimization of reaction parameters substantially enhanced feather hydrolysis efficiency. LC–MS/MS-based cleavage mapping further revealed preferential cleavage within flexible keratin regions enriched in small residues such as Ser and Thr.

Mutational analyses identified multiple structural elements required for catalytic activity, thermal stability, and autoproteolytic maturation, including extended loop regions, *β*-sheet elements, and the unusual catalytic-cavity residue Cys194. Together, these findings indicate that Fervitase employs a conserved thermitase-like catalytic scaffold supplemented by specialized structural features that enable efficient degradation of highly recalcitrant feather keratin under extreme conditions.

Given the large-scale generation of keratin-rich poultry waste worldwide, Fervitase represents a promising platform for sustainable feather valorization and high-temperature bioprocessing. The structural and mechanistic insights obtained in this study also provide a useful foundation for future engineering of thermophilic keratinases for industrial keratin bioconversion.

## Data Availability

The original contributions presented in the study are included in the article/Supplementary material, further inquiries can be directed to the corresponding authors.
