# Supplementary material for: Functional identification of a feather-degrading thermitase-like S8 serine protease from Fervidobacterium islandicum AW-1
Source: Front Microbiol. 2026 Aug 11;17:1886669. doi: 10.3389/fmicb.2026.1886669 (PMC13503562; doi:10.3389/fmicb.2026.1886669)
Supplement: Supplementary file 1 [file Supplementary_file_1.DOCX]

**Supplementary Information**

**Functional identification of a feather-degrading thermitase-like S8 serine protease from *Fervidobacterium islandicum* AW-1**

Ji Seob Kim^1,¶^, Jae-Yoon Sung^1,¶^, Hyeon-Su Jin^1^, Hakyoo Jeong^1^, Gaewon Nam^2,^*, and Dong-Woo Lee^1,^*

^1^Department of Biotechnology, Yonsei University, Seoul 03722, South Korea.

^2^Bio-Living Engineering Major, Global Leaders College, Yonsei University, Seoul 03722, South Korea.

**Running title:** Fervitase from *Fervidobacterium islandicum* AW-1

**Correspondence:**

**Gaewon Nam**

Bio-Living Engineering Major, Global Leaders College, Yonsei University, Seoul 03722, South Korea.

Tel.: +82-32-749-2650, E-mail: skarod@yonsei.ac.kr

**Dong-Woo Lee**

Department of Biotechnology, Yonsei University, Seoul 03722, Republic of Korea.

Tel.: +82-2-2123-2886, Fax.: +82-2-362-7265, E-mail: [leehicam@yonsei.ac.kr](mailto:leehicam@yonsei.ac.kr)

^¶^These authors contributed equally to this work.

Table S1. Protein sequences used for structural comparison

| **Enzyme** | **UniProt**  **accession** | **Source** | **Core region** | **References** | **RMSD (Å)** |
| --- | --- | --- | --- | --- | --- |
| Fervitase | A0AAI8CLQ9 | *Fervidobacterium islandicum* AW-1 | 135-439 | AF3 | 1.0 |
| Thermitase | P04072 | *Thermoactinomyces vulgaris* | 19-279 | PDB: 1THM | 0.801 |
| MtaKer | A0A2H4A2Y5 | *Meiothermus taiwanensis* WR-220 | 20-284 | PDB: 5WSL | 0.940 |
| DgeKer | Q1IZQ6 | *Deinococcus geothermalis* DSM 11300 | 152-420 | AF3 | 0.833 |
| GacK | A0A0A7GGG8 | *Geoglobus acetivorans* | 242-542 | AF3 | 0.833 |
| ProK | P06873 | *Parengyodontium album* | 125-383 | PDB: 1IC6 | 0.860 |
| Subtilisin | A0A0A7EAM0 | *Bacillus subtilis* | 100-362 | PDB: 4DWW | 0.748 |

Table S2. Primer design for mutagenesis

| **Primer** | **Sequence (5'→3')** |
| --- | --- |
| C194A-F | TACTCACGCTATAGGTACAGTTGCAGCTGTTACAAACAAC |
| C194A-R | GTACCTATAGCGTGAGTACCGTGGCTGACATC |
| S377A-F | GGCACAGCTATGGCAACACCACATGTTGCAG |
| S377A-R | TGTTGCCATAGCTGTGCCTTGCAAGAACATATATATGTCACC |
| ∆β-sheet-F | TTACAACTACTACCTCGACGTTGTTGCGCC |
| ∆β-sheet-R | CGTCGAGGTAGTAGTTGTAATTAGAATACGGTGCGCG |
| ∆long-loop-F | GAGACACAGTGCTGAGTACCACATGGACACC |
| ∆long-loop-R | TACTCAGCACTGTGTCTCCACCAGGCGC |
| ∆short-loop-F | AAGACAGCCACGTACGACTCTACGGGCTG |
| ∆short-loop-R | GTCGTACGTGGCTGTCTTTATCAATCTGTTTCTGATGGC |

**Table S3. List of recombinant feather keratin hydrolysates**

| **Sequence** | **Charges** | **PSMs^a^** | **Score^b^** | **Min PEP^c^** | **Mean m/z^d^** |
| --- | --- | --- | --- | --- | --- |
| TPPGTLHPT | 2 | 42 | 117.16 | 2.22E-44 | 460.745 |
| ASPVPRILTPFSGRLTPPGTLHPT | 3; 4 | 14 | 111.53 | 3.35E-202 | 778.344 |
| SCSNLCRPCGPTPLANSC | 2; 3 | 13 | 207.04 | 0 | 868.251 |
| SGRLTPPGTLHPTAM | 2; 3 | 12 | 121.9 | 3.28E-262 | 576.555 |
| SGRLTPPGTLHPT | 2; 3 | 11 | 101.3 | 6.74E-54 | 546.209 |
| SGRLTPPGTLHPTAMSC | 2; 3 | 9 | 119.25 | 2.13E-212 | 660.954 |
| NLCRPCGPTPLANSC | 2; 3 | 8 | 139.86 | 0 | 787.395 |
| SPVPRILTPFSGRLTPPGTLHPT | 3; 4 | 8 | 122.3 | 1.84E-254 | 712.779 |
| SNLCRPCGPTPLANSC | 2; 3 | 7 | 145.83 | 0 | 773.63 |
| PVPRILTPFSGRLTPPGTLHPT | 3; 4 | 7 | 140.97 | 0 | 757.434 |
| SAAVGSELSAQ | 1; 2 | 7 | 120.45 | 1.93E-227 | 583.003 |
| PRILTPFSGRLTPPGTLHPT | 3 | 7 | 104.88 | 5.03E-149 | 720.076 |
| SSAAVGSELSAQ | 2 | 6 | 138.98 | 0 | 553.77 |
| AAVGSELSAQ | 2 | 6 | 86.794 | 5.12E-67 | 466.738 |
| SNLCRPCGPTPLANSCNEPCVRQ | 3 | 6 | 145.44 | 0 | 896.402 |
| SNLCRPCGPTPLAN | 2 | 6 | 118.9 | 2.76E-207 | 778.869 |
| SSSAAVGSELSAQ | 2 | 6 | 110.53 | 2.03E-158 | 597.286 |
| SNLCRPCGPTPL | 2 | 6 | 104.22 | 1.65E-134 | 686.329 |
| SGRLTPPGTLHPTAM | 2; 3 | 6 | 103.34 | 5.83E-138 | 604.091 |
| QDSQVVIQPS | 1; 2 | 5 | 204.36 | 0 | 660.738 |
| PPGTLHPT | 2 | 5 | 129.2 | 1.75E-96 | 410.222 |
| VGSELSAQ | 1 | 5 | 116.54 | 4.61E-201 | 790.394 |
| LCRPCGPTPLANSC | 2; 3 | 5 | 158.05 | 0 | 748.473 |
| SGRLTPPGTLHPTAMSCS | 2; 3 | 5 | 91.457 | 1.17E-94 | 686.266 |
| PVPRILTPFSGRLTPPGTLHPTAM | 3; 4 | 5 | 46.578 | 5.16E-40 | 810.219 |
| LTPPGTLHPT | 2 | 4 | 83.753 | 1.14E-3 | 517.287 |
| ASPVPRILTPF | 2 | 4 | 94.309 | 1.21E-96 | 599.353 |
| SPVPRILTPFSGRLTPPGTLHPTAM | 3; 4 | 4 | 169.05 | 0 | 716.667 |
| SCSNLCRPCGPTPLAN | 2 | 4 | 164.13 | 0 | 902.4 |
| NLCRPCGPTPLANSCNEPCVRQ | 3 | 4 | 160.67 | 0 | 867.391 |
| NLCRPCGPTPLAN | 2 | 4 | 133.23 | 4.83E-288 | 735.353 |
| SCSNLCRPCGPTPL | 2 | 4 | 109.6 | 2.41E-159 | 809.86 |
| NLCRPCGPTPL | 2 | 4 | 107.47 | 2.83E-154 | 642.813 |
| PRILTPFSGRLTPPGTLHPTAM | 3 | 4 | 100.67 | 5.79E-137 | 787.435 |
| TPFSGRLTPPGTLHPT | 2; 3 | 4 | 96.414 | 1.4E-74 | 630.214 |
| AVGSELSAQ | 1; 2 | 4 | 71.922 | 5.21E-30 | 646.325 |
| AMSCSNLCRPCGPTPLANSC | 2; 3 | 4 | 53.448 | 6.23E-18 | 939.31 |
| VVVTLPGPILS | 1; 2 | 3 | 188.76 | 0 | 730.124 |
| GRLTPPGTLHPT | 2; 3 | 3 | 134.59 | 5.21E-119 | 554.644 |
| SGRLTPPGTL | 2 | 3 | 122.13 | 4.33E-59 | 499.785 |
| TPPGTLHPTAM | 2 | 3 | 110.12 | 2.26E-36 | 569.782 |
| IKASPVPRIL | 2 | 3 | 90.657 | 1.00E-3 | 547.358 |
| TLPGPILS | 1; 2 | 3 | 128.86 | 8.28E-86 | 664.732 |
| SPVPRILTPF | 2 | 3 | 90.657 | 4.19E-40 | 563.835 |
| SCSNLCRPCGPTPLANSCNEPCVRQ | 3 | 3 | 175.28 | 0 | 978.756 |
| GHSHSSIKASPVPRILTPF | 3; 4 | 3 | 133.71 | 0 | 564.926 |
| LCRPCGPTPLAN | 2 | 3 | 115.78 | 6.72E-203 | 678.332 |
| SPVPRILTPFSGRLTPPGTLHPTAM | 4 | 3 | 110.75 | 7.89E-212 | 665.615 |
| AMSCSNLCRPCGPTPLAN | 2 | 3 | 97.69 | 5.84E-66 | 1003.439 |
| NEPCVRQCQDSQVVIQPS | 2 | 3 | 92.856 | 2.99E-79 | 1072.497 |
| LCRPCGPTPL | 2 | 3 | 92.439 | 5.6E-96 | 585.792 |
| NLCRPCGPTPLANSCNEPCVRQC | 3 | 3 | 78.413 | 9.17E-73 | 920.735 |
| PVPRILTPF | 2 | 3 | 75.109 | 2.93E-40 | 520.319 |
| SGRLTPPGTLHPTAMS | 3 | 3 | 35.329 | 3.76E-2 | 541.615 |
| SGGFGFGGL | 1 | 2 | 160.17 | 6.91E-256 | 798.378 |
| VVTLPGPILSS | 2 | 2 | 143.12 | 2.48E-171 | 541.826 |
| GGFGFGGL | 1 | 2 | 102.29 | 1.36E-14 | 711.346 |
| AVGSELSAQGVA | 2 | 2 | 93.992 | 1.09E-40 | 544.783 |
| AVGSELSAQGV | 2 | 2 | 85.642 | 7.96E-30 | 509.264 |
| LCRPCGPTPLANSCNEPCVRQ | 3 | 2 | 170.06 | 0 | 829.377 |
| GVAISSGGFG | 1 | 2 | 164.24 | 0 | 851.426 |
| AVGSELSAQGVAIS | 2 | 2 | 155.84 | 0 | 644.841 |
| GPTPLANSC | 2 | 2 | 148.33 | 0 | 458.713 |
| IKASPVPRILTPF | 2; 3 | 2 | 118.28 | 1.06E-156 | 600.12 |
| AMSCSNLCRPCGPTPL | 2 | 2 | 117.2 | 4.21E-212 | 910.899 |
| SCSNLCRPCGPTPLANS | 2 | 2 | 117.07 | 2.05E-212 | 945.916 |
| AAVGSELSAQGVAISS | 2 | 2 | 112.85 | 4.94E-187 | 723.875 |
| SGGFGFGGLGCFN | 2 | 2 | 101.46 | 6.58E-135 | 638.774 |
| CSNLCRPCGPTPLANSC | 2 | 2 | 93.959 | 4.76E-104 | 982.416 |
| PFSGRLTPPGTLHPT | 2; 3 | 2 | 85.837 | 2.04E-55 | 658.022 |
| SGRLTPPGTLHPTAMSC | 3 | 2 | 84.859 | 1.03E-73 | 600.291 |
| TLPGPILSSFPQNTAVGSS | 2 | 2 | 80.17 | 1.91E-65 | 936.989 |
| TAVGSSSSAAVGSELSAQ | 2 | 2 | 72.731 | 1.27E-41 | 804.889 |
| LCRPCGPTPLANS | 2 | 2 | 67.334 | 3.44E-22 | 721.848 |
| SNLCRPCGPTPLANSCN | 2 | 2 | 64.55 | 2.25E-26 | 959.422 |
| CRPCGPTPL | 2 | 2 | 57.559 | 1.14E-08 | 529.25 |
| NLCRPCGPTPLANS | 2 | 2 | 57.174 | 7.11E-10 | 778.869 |
| LCRPCGPTPLANSCNEPCVRQC | 3 | 2 | 57.009 | 1.75E-21 | 882.72 |
| SCSNLCRPCGPTPLANSCN | 2; 3 | 2 | 30.956 | 2.64E-4 | 902.629 |
| KASPVPRIL | 2 | 1 | 138.54 | 2.3E-127 | 490.816 |
| LPGPILSS | 1 | 1 | 130.01 | 4.46E-86 | 783.461 |
| VVTLPGPILS | 2 | 1 | 113.89 | 1.43E-34 | 498.31 |
| VTLPGPIL | 1 | 1 | 88.201 | 6.29E-3 | 809.513 |
| FPQNTAVG | 1 | 1 | 77.282 | 9.69E-3 | 833.415 |
| DSQVVIQPS | 2 | 1 | 69.825 | 4.15E-3 | 486.753 |
| RLTPPGTLHPT | 2 | 1 | 67.897 | 4.88E-3 | 595.338 |
| VVTLPGPIL | 2 | 1 | 64.776 | 4.40E-3 | 454.794 |
| GRLTPPGTL | 2 | 1 | 63.565 | 3.71E-3 | 456.269 |
| TLPGPILSS | 2 | 1 | 60.76 | 2.70E-3 | 442.758 |
| ISSGGFGFGG | 1 | 1 | 54.205 | 3.38E-3 | 885.41 |
| VVVTLPGPIL | 1 | 1 | 45.718 | 7.94E-3 | 1007.65 |
| GVAISSGGF | 1 | 1 | 153.38 | 0 | 794.404 |
| TPFSGRLTPPGTLHPTAM | 3 | 1 | 148.41 | 0 | 627.661 |
| CRPCGPTPLANSC | 2 | 1 | 147.95 | 0 | 745.321 |
| GVAISSGGFGFGG | 1 | 1 | 132.76 | 7.63E-288 | 1112.537 |
| SGGFGFGGLGCFNGWRARYPC | 3 | 1 | 131.48 | 0 | 775.014 |
| TVVVTLPGPILS | 2 | 1 | 118.5 | 2.36E-203 | 598.368 |
| PFSGRLTPPGTLHPTAM | 2 | 1 | 116.24 | 2.64E-212 | 890.464 |
| AAVGSELSAQGVAI | 2 | 1 | 115.71 | 1.1E-206 | 636.843 |
| SQVVIQPS | 1 | 1 | 104.44 | 1.23E-99 | 857.473 |
| PPGTLHPTAM | 2 | 1 | 91.9 | 6.34E-96 | 511.26 |
| NTAVGSSSSAAVGSELSAQ | 2 | 1 | 86.756 | 4.68E-80 | 861.911 |
| AVGSELSAQGVAISSGG | 2 | 1 | 80.979 | 2.75E-60 | 745.378 |
| SPVPRILTPFSGRL | 3 | 1 | 77.776 | 1.74E-55 | 513.972 |
| SSSSAAVGSELSAQ | 2 | 1 | 74.841 | 9.37E-43 | 640.802 |
| AAVGSELSAQGV | 2 | 1 | 70.69 | 5.47E-30 | 544.783 |
| GPTPLANSCNEPCVRQ | 2 | 1 | 66.262 | 2.68E-26 | 900.412 |
| ASPVPRIL | 2 | 1 | 62.714 | 8.68E-16 | 426.769 |
| ASPVPRILTPFSGRLTPPG | 3 | 1 | 61.167 | 4.64E-24 | 655.042 |
| AMSCSNLCRPC | 2 | 1 | 59.8 | 1.96E-13 | 678.27 |
| GTLHPTAM | 1 | 1 | 55.441 | 6.82E-10 | 827.408 |
| ASPVPRILTPFSGRL | 3 | 1 | 53.828 | 2.24E-32 | 537.651 |
| SAAVGSELSAQGVAI | 2 | 1 | 52.49 | 2.65E-09 | 680.359 |
| GVAISSGGFGFGGLGCF | 2 | 1 | 51.445 | 4.29E-15 | 795.374 |
| SSAAVGSELSAQGVAI | 2 | 1 | 50.292 | 6.29E-09 | 723.875 |
| SAAVGSELSAQGVAIS | 2 | 1 | 48.998 | 1.71E-6 | 723.875 |
| GGFGFGGLGCFNGWRARYPC | 3 | 1 | 47.568 | 8.25E-08 | 746.004 |
| AMSCSNLCRPCGPTPLANSC | 3 | 1 | 45.221 | 1.76E-7 | 756.981 |
| SSAAVGSELSAQGVAIS | 2 | 1 | 44.847 | 5.95E-5 | 767.391 |
| AMSCSNLCRPCGPTPL | 2 | 1 | 43.68 | 1.44E-3 | 918.897 |
| GVAISSGGFGFG | 2 | 1 | 43.246 | 3.73E-2 | 528.261 |
| TPPGTLHPTAM | 2 | 1 | 41.029 | 8.19E-2 | 561.784 |
| FSGRLTPPGTLHPTAM | 3 | 1 | 40.801 | 3.18E-7 | 561.628 |
| CSNLCRPCGPTPL | 2 | 1 | 40.715 | 8.19E-2 | 766.344 |
| SIKASPVPRILTPF | 3 | 1 | 40.552 | 7.40E-3 | 509.308 |
| LTPFSGRLTPPGTLHPTA | 3 | 1 | 40.085 | 3.99E-4 | 621.676 |
| CSNLCRPCGPTPLAN | 2 | 1 | 39.148 | 2.20E-2 | 858.884 |
| SGRLTPPGTLHPTAMSCSNL | 3 | 1 | 38.271 | 1.05E-3 | 699.679 |
| SGRLTPPGTLHPTAMSCSN | 3 | 1 | 37.624 | 1.71E-3 | 661.984 |
| TPFSGRLTPPGTLHPTAMSC | 3 | 1 | 36.523 | 1.90E-3 | 710.015 |
| TVVVTLPGPILSSFPQNTAVGSSSS | 2 | 1 | 34.82 | 7.77E-3 | 1223.147 |
| AVGSELSAQGVAI | 2 | 1 | 33.263 | 7.03E-2 | 601.325 |
| GRLTPPGTLHPTAM | 3 | 1 | 32.734 | 1.53E-3 | 483.594 |
| SLQALGHSHSSIKASPVPRILTPF | 4 | 1 | 32.391 | 3.08E-2 | 636.608 |
| TLPGPILSSFPQNTAVGSSSSA | 2 | 1 | 32.275 | 3.41E-2 | 1059.539 |
| GVAISSGGFGFGGLG | 2 | 1 | 30.564 | 2.21E-2 | 641.825 |
| ILTPFSGRLTPPGTLHPTAM | 3 | 1 | 30.416 | 4.91E-4 | 703.05 |
| SNLCRPCGPTPLANS | 2 | 1 | 29.341 | 3.81E-2 | 822.385 |
| GVAISSGGFGFGGLGCFN | 2 | 1 | 29.317 | 8.85E-5 | 852.396 |
| FSGRLTPPGTLHPT | 3 | 1 | 25.53 | 5.42E-2 | 494.268 |
| TAVGSSSSAAVGSEL | 2 | 1 | 25.238 | 5.82E-2 | 661.825 |
| RLTPPGTLHPTAM | 2 | 1 | 24.855 | 5.79E-2 | 696.377 |
| SSSAAVGSELSAQGVAI | 2 | 1 | 24.853 | 8.40E-2 | 767.391 |
| NLCRPCGPTPLANSCN | 2 | 1 | 22.474 | 2.90E-2 | 915.906 |

^a^ PSM, peptide-spectrum matches; ^b^score, Best Andromeda score; ^c^PEP, posterior error probability; ^d^m/z, mass to charge ratio.
